# In Silico Drug Repurposing Studies for the Discovery of Novel Salicyl-AMP Ligase (MbtA)Inhibitors

**DOI:** 10.3390/pathogens12121433

**Published:** 2023-12-09

**Authors:** Gourav Rakshit, Abanish Biswas, Venkatesan Jayaprakash

**Affiliations:** Department of Pharmaceutical Sciences & Technology, Birla Institute of Technology, Mesra, Ranchi 835215, Jharkhand, India; gouravr16@gmail.com (G.R.); abanish37@gmail.com (A.B.)

**Keywords:** Mycobacterium, antibiotic resistance, drug repurposing, MbtA, siderophores, molecular docking, molecular dynamics, PCA analysis

## Abstract

Tuberculosis (TB) continues to pose a global health challenge, exacerbated by the rise of drug-resistant strains. The development of new TB therapies is an arduous and time-consuming process. To expedite the discovery of effective treatments, computational structure-based drug repurposing has emerged as a promising strategy. From this perspective, conditionally essential targets present a valuable opportunity, and the mycobactin biosynthesis pathway stands out as a prime example highlighting the intricate response of *Mycobacterium tuberculosis* (Mtb) to changes in iron availability. This study focuses on the repurposing and revival of FDA-approved drugs (library) as potential inhibitors of MbtA, a crucial enzyme in mycobactin biosynthesis in Mtb conserved among all species of mycobacteria. The literature suggests this pathway to be associated with drug efflux pumps, which potentially contribute to drug resistance. This makes it a potential target for antitubercular drug discovery. Herein, we utilized cheminformatics and structure-based drug repurposing approaches, viz., molecular docking, dynamics, and PCA analysis, to decode the intermolecular interactions and binding affinity of the FDA-reported molecules against MbtA. Virtual screening revealed ten molecules with significant binding affinities and interactions with MbtA. These drugs, originally designed for different therapeutic indications (four antiviral, three anticancer, one CYP450 inhibitor, one ACE inhibitor, and one leukotriene antagonist), were repurposed as potential MbtA inhibitors. Furthermore, our study explores the binding modes and interactions between these drugs and MbtA, shedding light on the structural basis of their inhibitory potential. Principal component analysis highlighted significant motions in MbtA-bound ligands, emphasizing the stability of the top protein–ligand complexes (PLCs). This computational approach provides a swift and cost-effective method for identifying new MbtA inhibitors, which can subsequently undergo validation through experimental assays. This streamlined process is facilitated by the fact that these compounds are already FDA-approved and have established safety and efficacy profiles. This study has the potential to lay the groundwork for addressing the urgent global health challenge at hand, specifically in the context of combating antimicrobial resistance (AMR) and tuberculosis (TB).

## 1. Introduction

For centuries, tuberculosis (TB) has haunted humanity as an infectious disease caused by the lethal pathogen *Mycobacterium tuberculosis* (Mtb). Today, this bacterium has escalated into a global pandemic that wreaks havoc worldwide [1]. The World Health Organization (WHO) released a 2021 report stating that TB caused 1.6 million deaths (including 187,000 people with HIV) and affected more than 10.6 million people worldwide in 2021, signifying its dire impact on mortality and morbidity rates globally [2]. As per the WHO’s latest estimates, one in four individuals currently carry an established TB infection. Mtb is a severe bacterial infection that primarily affects the lungs, but can also affect other parts of the body. Its ability to transition between respiring and non-respiring conditions without losing viability as a result of various enzymatic reactions leads to challenges in tackling TB infection [3]. It is a global health issue that requires improved living conditions, better access to healthcare, and increased awareness and prevention efforts to combat its spread. The United Nations has been actively involved in efforts to combat tuberculosis (TB) globally. The UN’s Sustainable Development Goals include a target to end the TB epidemic by 2030 [2].

**Figure 1:**
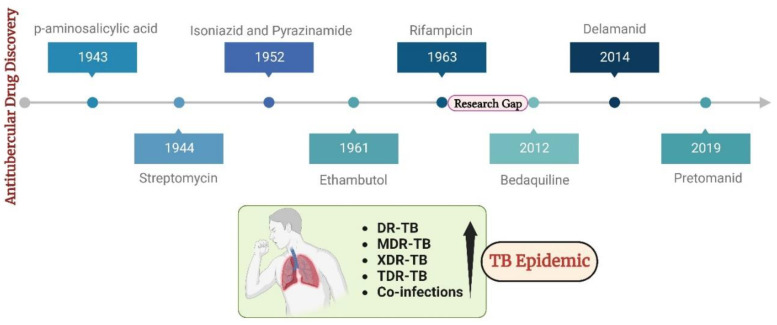
The timeline of drug discovery in TB alongside the present situation.

The escalation in global infections has led to drug discovery and development becoming central areas of focus within the scientific community. Antitubercular drug discovery began with the discovery of p-amino salicylic acid (1943), streptomycin (1944), isoniazid and pyrazinamide (1952), ethambutol (1961), and rifampicin (1963). Following this, there was a forty-year research pause, primarily due to a lack of worldwide financing, resistant existing drug targets, unviable new drug targets, and clinical trial failures of novel medications. In an effort to mitigate the atrocities associated with infectious TB, a few clinically approved drugs were launched: bedaquiline (2012), delamanid (2014), and pretomanid (2019) [4]. These drugs continue to be the only choice of treatment for drug-resistant TB to date. Recently, TB has become an epidemic due to the surge in numerous resistance cases, particularly DR-TB, MDR-TB, XDR-TB, and TDR-TB cases [5,6]. These drug-resistant cases seem to be potentially incurable with available therapies. Moreover, the existence of co-infections in the pre- and post-COVID eras has worsened the current scenario in tacking TB [7,8,9]. These factors necessitate the search for novel antitubercular agents acting via novel mechanisms of action on novel targets. This could open avenues for tackling drug resistance. Figure 1 outlines the timeline of drug discovery in TB alongside the present situation.

**Figure 2:**
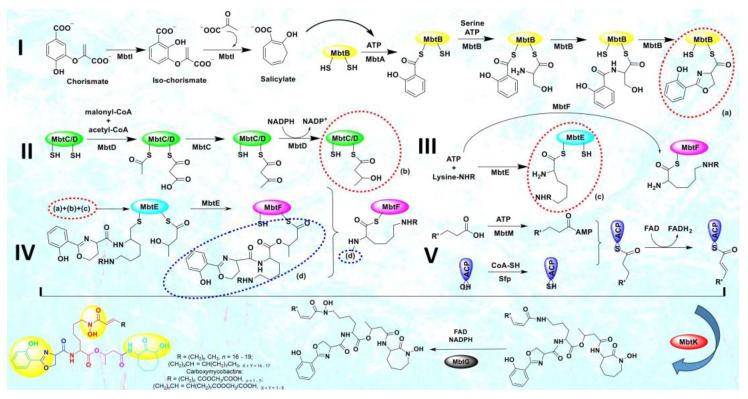
The biosynthetic pathway of mycobacterial siderophores, mycobactins, and carboxymycobactins.

**Figure 3:**
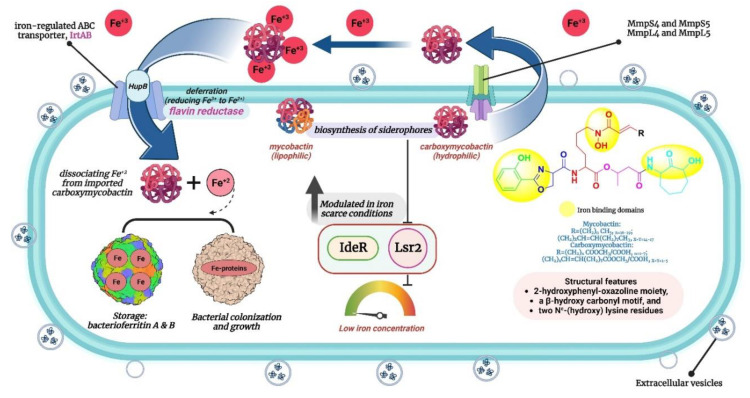
Mtb’s iron response: when iron is scarce, Mtb boosts genes for siderophore synthesis.

In this sense, CET (conditionally essential target)-based drug design offers an excellent opportunity [10]. This encompasses focusing on a process that is conditionally necessary for mycobacteria, supporting their enzymatic functions and aiding in bacterial colonization, proliferation, and growth. Mycobacteria rely on the essential process of iron acquisition, vital for their biochemical machinery, from human host sources [11,12,13]. Although serum iron is scarce (10^−24^ M), abundant iron is found in extracellular bound form. Mycobacteria have evolved various mechanisms to counter iron scarcity, notably synthesizing, secreting, and re-uptaking small molecules termed mycobactins/siderophores/iron chelators [14,15]. This siderophore machinery becomes active under iron-deficient conditions, a critical factor in tuberculosis pathogenesis [16]. The mycobactin biosynthesis pathway involves non-ribosomal peptide synthetase and polyketide synthase (NRPS-PKS) assembly chains [17]. Building blocks like salicylate and modified lysine are synthesized externally and linked to NRPS-PKS enzymes, as shown in Figure 2. Amino acids are added to growing mycobactin molecules. Mycobactins are hexadentate ligands with a tripodal structure, comprising an *o*-hydroxyphenyloxazoline system linked to an acylated hydroxylysine residue attached to a 3-hydroxybutyric acid unit, further tethered to a cyclized hydroxylysine, forming a seven-membered lactam ring [18]. Mtb maintains iron homeostasis, regulating uptake, utilization, and storage [19]. Under iron stress, genes for siderophore biosynthesis (mbt), export (MmpL4/5-MmpS4/5), and import (IrtAB) are upregulated [20]. Siderophore synthesis involves proteins like HupB, IdeR, and Lsr2. Chelating agents/mycobactins are released in the intracellular space. Carboxymycobactins are exported via the inner-membrane transporters MmpL4/5 with MmpS4/5 adaptors. Mycobactin is exported via extracellular vesicles. Carboxymycobactins transfer chelated Fe^3+^ to mycobactin via HupB, acting as a receptor [21]. Once iron is chelated, it is internalized as a ferri-carboxymycobactin/ferri-mycobactin complex and imported independently via iron-regulated ABC transporter IrtAB [22]. IrtAB is a unique inner membrane heterodimer coupling Fe^3+^-carboxymycobactin import and iron assimilation through IrtA’s cytosolic domain, functioning as a flavin reductase, converting Fe^3+^ to Fe^2+^ [23]. Mtb stores iron in bacterioferritin (BfrA) and ferritin (BfrB) to maintain iron balance [24,25]. Iron toxicity is prevented by the type VII Esx-3 secretion system, although its precise role in iron uptake is unclear [26,27]. Carboxymycobactins and mycobactin, once deferrated, are recycled and exported via MmpL4/5. From this machinery, it is evident that the absence of the mycobactin gene inhibits growth in iron deficiency, highlighting the significance of the mbt gene cluster [28]. Figure 3 presents an overview of the above-mentioned process.

Until now, researchers have explored merely four enzymes—MbtI, MbtA, MbtM, and PPTase—within the mycobactin biosynthesis pathway as potential drug targets against tuberculosis, and the advances have been published by our team in the form of a review [4]. Our primary focus lies on MbtA (salicyl-AMP ligase) due to its role in catalyzing the initial step of mycobactin biosynthesis and its critical role in bacterial growth and virulence. In the mycobactin megasynthase gene cluster, a crucial bimodular system comprising salicyl-AMP ligase (MbtA) and phenyloxazoline synthetase (MbtB) drives the production of the heterocyclic oxazoline segment within the mycobactin structure. MbtA plays a pivotal role in initiating mycobactin biosynthesis through two sequential reactions, first, by catalyzing the activation of salicylic acid, forming salicyl-adenosine monophosphate (Sal-AMP), and then, by transferring Sal-AMP to the phosphopantetheinylation domain of MbtB. MbtB, an acyl carrier protein, acts as the adjoining enzyme to MbtA in the NRPS-PKS cluster. The ensuing MbtB-bound salicyl-thioester is condensed with ser—anchored to MbtB’s C-terminal phosphopantetheinylation site—resulting in the formation of an amide. Through several subsequent steps, this amide is cyclized to generate the 2-hydroxyphenyloxazoline core structure of mycobactins. This MbtA-MbtB bimodular system is an indispensable and conserved element of the biochemical machinery employed by pathogenic mycobacterial species to survive in iron-deficient conditions within host macrophages, making it a promising target for therapeutic intervention [29]. Despite the existence of a few inhibitors (nucleoside and non-nucleoside) targeting MbtA [7,30,31], the quest to discover a more suitable inhibitor persists, and furthermore, the study of MbtA inhibitors is currently in its preliminary stages.

We are pioneering drug repurposing for this target (MbtA) to aid global scientific efforts against tuberculosis and antimicrobial resistance. Our focus centers on repurposing FDA-approved drug compounds that target mycobacterial MbtA, a critical enzyme within the mycobactin biosynthetic pathway. Through the utilization of structure-based drug design and virtual screening, we systematically evaluated the entire molecular library of FDA-reported drugs to discern their binding and molecular interactions. This extensive assessment allowed us to gather valuable insights into the selective binding of FDA drugs to MbtA, enabling the assessment of their potential as therapeutic agents. To delve deeper, molecular dynamics simulations were employed to probe the molecular underpinnings of inhibition, exploring the binding modes and the stability of the complex formed between inhibitors and the target enzyme. These in silico analyses provide an initial and comprehensive understanding of the binding mechanisms and the potential of FDA molecules as MbtA inhibitors. This approach provides deeper insights into the individual amino acids’ contributions to ligand binding and the presence of crucial protein–ligand interactions during simulations, along with frame analysis and PCA analysis. Such findings hold the promise of guiding the development of novel therapeutic agents that target MbtA, thereby paving the way for potential applications in combatting resistance challenges. The outcomes of this study hold potential for informing the reformation and assessment of new potential medications to combat tuberculosis and antimicrobial resistance. Moreover, these findings contribute to our broader comprehension of inhibitory mechanisms. Insights gained from this study would increase our preparedness against TB, which can evolve into a deadly pandemic. Our team also has plans to carry out biological evaluations of the top-ranking hit compounds in order to assess their inhibitory activity against MbtA and as efflux pump modulators. These efforts would also contribute to addressing the global challenge of coinfections.

## 2. Materials and Methods

### 2.1. System Specifications and Software Employed

All molecular simulations were performed on a workstation with the following specifications: operating system: Ubuntu 22.04 LTS, 64-bit; processor: Intel^®^ Core™ i5-12400 CPU@2.30 GHz processor; RAM: 16 GB; and graphics: 8 GB Nvidia GeForce RTX 3050 GPU. The software employed for the study included the following: (i) The ligand database (FDA-Approved Drugs) for carrying out virtual screening was downloaded from the web server of https://zinc.docking.org/, (accessed on 31 May 2023 [32,33]. The file named *fda.smi* was saved for further molecular simulation studies. (ii) The co-crystallized protein structure of MbtA was obtained from the Protein Data Bank (PDB) (https://www.rcsb.org/; accessed on 31 May 2023) and Alpha Fold database (https://alphafold.ebi.ac.uk/; accessed on 31 May 2023) [34,35]. (iii) Molecular docking and virtual screening were performed using AutoDock-GPU in the Google Colab web server (https://colab.research.google.com/; accessed on 31 May 2023). The best conformers of the ligands were generated using an in-house Python script, *write_largest_cluster_ligand.py*, located in AutoDock 4.2.6 [36]. A summary of the virtual screening was generated using an in-house Python script: *summarize_docking.py*. (iv) Protein–ligand complexes (PLCs) were generated using AutoDock 4.2.6 [37]. To carry out the molecular dynamics simulations (MDS), GROMACS ( GROningen MAchine for Chemical Simulations) version 2022.4 was utilized [38]. (v) All 2D and 3D visualizations were carried out using PyMOL (molecular visualization system) and LigPlot+ (academic version) [39,40].

### 2.2. Ligand Preparation

To conduct the virtual screening by molecular docking, the *fda.smi* file containing the details of ligands downloaded from the ZINC database (https://zinc.docking.org/; accessed on 31 May 2023) was used [41]. Using Open Babel: The Open-Source Chemistry Toolbox, v. 2.4.0, the ligand library was converted to individual *ligand.pdb* file format [42]. Then, the ligands (*ligand.pdb*) were imported into AutoDock-GPU in Google Colab, and their energy was minimized. This process aided in the determination of bond orders and the addition of hydrogens to the ligands required for the docking studies. The individual output files (*ligand.pdbqt*) containing the best conformations of the ligands were used for further docking-based screening studies.

### 2.3. Protein Preparation

The protein structure of salicyl-AMP ligase/salicyl-S-ArCP synthetase (565 amino acids) was obtained from the Alpha Fold Protein Structure Database (https://alphafold.com/; accessed on 31 May 2023). The source organism was *Mycobacterium tuberculosis* (strain ATCC 25618/H37Rv) with protein code P71716 (MBTA_MYCTU). The PDB-BLAST search outcomes demonstrated that Mtb-MbtA shares sequence identity with related proteins, such as the crystal structure of DhbE bound with 2,3-dihydroxybenzoic acid (DHB)-adenylate (PDB code 1MDB) from the *Bacillus subtilis* and *Mycobacterium smegmatis* MbtA apo structure (PDB ID: 5KEI) [43,44]. The active site of the MbtA protein was identified by analogy with these homologous structures and was prepared using the protein tab in the AutoDock 4.2.6 program by MGLTools 1.5.6 for docking. The preparation steps involved the (i) deletion of water molecules, (ii) addition of polar hydrogens, (iii) merging non-polar hydrogens, (iii) specification of AD4 atom type, and (iv) addition of Gasteiger charges. Finally, the protein was energy-minimized and saved in *protein.pdbqt* format for protein–ligand docking.

### 2.4. Identification of Binding Site and Receptor Grid Generation

Locating the active site is an essential parameter for protein–ligand docking. The probable active site in the model was identified by aligning it with the template of the ligand-bound DHB adenylating enzyme DhbE (PDB ID: 1MDB). This enabled a comparison of the active site residues in both structures, which was essential for conducting the following docking studies. The grid was generated based on the position of the co-crystallized ligand present in the active site of the selected protein (PDB: 1MDB). Subsequently, the MbtA protein was aligned with this ligand’s position, and a grid box was formed around the centroid of the co-crystallized ligand. The receptor atoms’ van der Waals radius was adjusted to 1.00 Å, along with a partial atomic charge of 0.25. This approach was chosen due to the conserved nature of the active site, given the homologous structure of both proteins. An input file (*protein.gpf*) was generated, and AutoGrid executable file was run to generate an output (*protein.glg*) file. The exact grid coordinates were further used in virtual screening, as presented in Table 1.

### 2.5. Docking-Based Virtual Screening Studies

Prior to commencing virtual screening, validation of the docking program was performed. The co-crystallized ligand of 1MDB was re-docked into the active site of the MbtA protein (homology). Subsequently, the root mean square deviation (RMSD) was calculated. Then, docking-based screening of the FDA-reported molecules (*fda.smi*) was performed in the active site of the MbtA receptor (by analogy with the related structure with the PDB ID: 1MDB) to evaluate the binding modes and inhibitory profile in AutoDock-GPU in Google Colab [45]. Similar grid parameters were employed to those used in validation studies. The script is available as *AutoDock_GPU_VJ.ipynb* on GitHub (https://github.com/; accessed on 31 May 2023). The following docking parameters were established: a population size of 150 ensured accuracy within a reasonable computation time, a maximum of 2,500,000 energy evaluations were conducted to explore ample conformational space, and the genetic algorithms were capped at 27,000 generations for convergence. A total of 200 runs were executed to achieve reliable sampling of the conformational space. A visual representation (2D and 3D) of the docked structures was achieved using LigPlot+ v.2.2 and PyMOL.

### 2.6. Molecular Dynamics Studies

Utilizing the GROMACS package (version 2022.4, single precision, GPU enabled) along with the CHARMM force field, molecular dynamics simulations were performed [38]. The optimization of the MbtA protein structure involved the removal of water molecules, the addition of hydrogen atoms, and energy minimization through the steepest descent algorithm (5 ns). TIP3P water molecules were employed to solvate the system within a cubic box, maintaining a protein–box-edge distance of 10 Å. Neutralization of the system was achieved by introducing counterions (Na^+^ or Cl^−^) utilizing the *gmx_genion* module. Simulations were executed within an NPT and NVT ensemble, adhering to periodic boundary conditions, operating at a temperature of 300 K and a pressure of 1 atm. Long-range electrostatic interactions were computed by the particle mesh Ewald method (cutoff distance: 12 Å) [46]. The simulations were run for 300 ns with coordinates and velocities saved at 10 ps intervals for subsequent analysis. The *md300.xtc*, *md300.tpr*, and *md300.edr* files generated were employed to generate RMSD, RMSF, ROG, and SASA data for analysis. Based on the stability trends observed in the RMSD graph of the protein–ligand complexes, a particular frame was selected for in-depth analysis. This frame serves as the foundation for a more comprehensive discussion on the essential residues involved in ligand binding, with a focus on H-bonds.

### 2.7. Principal Component Analysis (PCA)

Principal component analysis (PCA) was utilized to highlight the primary motions observed in protein trajectories (MD simulation) when bound to ligands. This analysis reveals that protein dynamics involve changes in molecular structure and conformation over time. PCA, a robust multivariate statistical technique, effectively reduces the dimensionality needed to describe protein dynamics through a systematic decomposition process, prioritizing observed motions from the largest to the smallest spatial scales [47]. The application of PCA to protein trajectories is referred to as essential dynamics (ED), aiming to extract the “essential” motions from sampled conformations. In practical terms, substantial large-scale motions can hinder the discernment of smaller-scale motions due to their significant amplitude in atomic displacements. It is noteworthy that these larger-scale motions often hold the utmost biological relevance in the realm of protein dynamics. Herein, PCA analysis was performed for all seven PLCs under study to explore molecular motion through molecular dynamics (MD) trajectories. To understand the different conformations of a given protein, relating both structure and dynamics, a trajectory was obtained by concatenating the molecular dynamics trajectories of the individual conformations. To eliminate the translational and rotational motion of the molecule, a “least squares fit” to the reference structure was applied. This procedure involved obtaining a covariance matrix by linearly transforming the Cartesian coordinate space. The matrix was then diagonalized to yield a set of eigenvectors that effectively represent the molecule’s motion. Each eigenvector’s associated eigenvalue indicated its energy contribution to the motion. Moreover, projecting the trajectory onto an eigenvector provided insights into the “time-dependent motions” exhibited by specific components within distinct vibrational modes. The temporal average of this projection helped discern the contribution of atomic vibrational components to this coordinated motion. To generate the eigenvectors and eigenvalues for the trajectory, the covariance matrix was computed and diagonalized using the integrated GROMACS utility “*g_covar*.” Additionally, the “*g_anaeig*” tool was employed for examining and visualizing the eigenvectors [48]. In summary, for PCA analysis, a combination of techniques and tools, including least squares fitting, “*g_covar*,” and “*g_anaeig*,” integrated within GROMACS, were employed for the comprehensive analysis and visualization of molecular dynamics.

## 3. Results

### 3.1. Molecular Docking Studies on MbtA

#### 3.1.1. Validation of Docking Procedure

**Figure 4:**
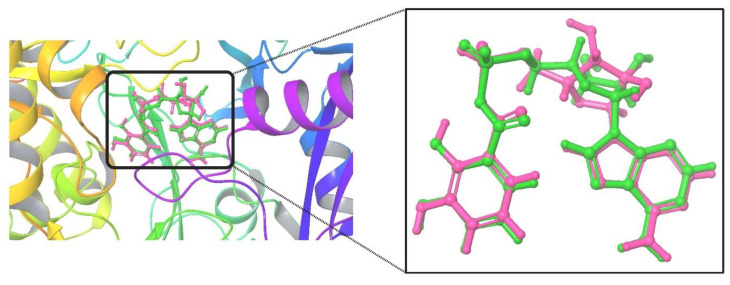
The superimposed overlay conformation of the docked internal ligand (green) concerning its crystallized conformation (pink) obtained from the co-crystallized complex structure.

The studies of the crystal structure of MbtA were validated through redocking (internal ligand: DHB-Adenylate), which resulted in a binding energy of −6.41 kcal/mol, a K_i_ of 20.06 µM, and a reference RMSD of 1.52 Å. Small fluctuations in RMSD (0–3 Å) are acceptable for small globular macromolecules. Figure 4 presents the overlay conformation of the internal ligand with its co-crystallized conformation.

#### 3.1.2. Virtual Screening of FDA-Reported Library through Molecular Docking

**Figure 5:**
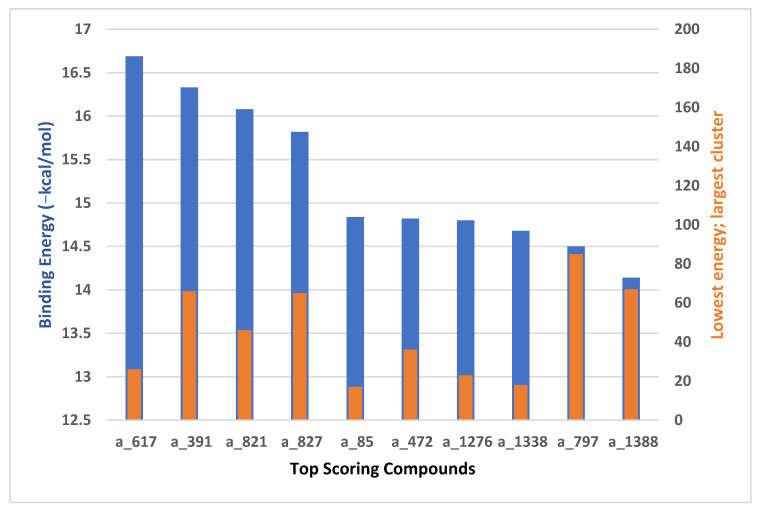
Graph comparing the lowest binding energies/docking scores and the number of conformations in the cluster (largest cluster) for the top-scoring docked molecules in MbtA protein’s active site.

This study utilizes the principles of structure-based drug design to identify potential molecules to tackle TB and AMR (antimicrobial resistance). Furthermore, we utilized the structure of MbtA to identify suitable binding agents with established safety profiles. For this purpose, FDA-approved drugs served as an ideal starting point, enabling the repurposing of safe and approved medications for combating tuberculosis. The virtual screening uncovered numerous molecules with favorable negative binding energies, including essential parameters such as docking scores, ligand efficiency, lipophilicity, hydrogen bonding interactions for each drug, and various other vital parameters. However, we opted to choose the top 10 hit molecules (results detailed in Table 2 and Table 3) with the highest negative binding energies. A higher negative binding energy indicates a stronger affinity for the active site. A bar graph representation of the same is shown in Figure 5 for a better understanding. Upon scrutinizing the molecules with the highest rankings, a notable observation emerged: among the top 10 molecules, four molecules (a_391: Saquinavir, a_85: Ritonavir, a_472: Lopinavir, and a_1276: Indinavir) were found to be protease inhibitors widely employed in antiviral therapies, notably recognized for their effectiveness against HIV and with potential to be used as boosters to antiviral therapies; three molecules possessed anticancer properties (a_821: Carfilzomib, a_1338: Venetoclax, and a_1388: Neratinib); one molecule was a CYP450 inhibitor (a_617: Cobicistat); one was an ACE inhibitor (a_827: Candesartan); and one molecule was a leukotriene antagonist (a_797: Zafirlukast (Accolate)). Thus, these molecules, stemming from various significant pharmacological classes, could potentially pave the way for exploring combination drug development aimed at tackling dual coinfections. To gain insights into the crucial interacting motifs, the ten-compound set was divided into groups on the basis of biological activity.

**Interaction analysis of the top-scoring compounds obtained through virtual screening:** The interpretation of molecular docking outcomes relies on specific descriptors, including binding energy, electrostatic energy, hydrogen bonding, van der Waals energy, and solvation energies [57]. Binding energy serves as a quantitative measure to compare and prioritize different ligands or potential drug candidates. It represents the overall energetic change associated with forming a stable complex between the ligand and receptor. Ligands with lower binding energies indicate a more favorable, strong, and specific interaction, suggesting a stronger binding affinity with the target receptor and vice versa. Binding energies are often decomposed into various energy terms, as mentioned above, and analyzing these individual components provides a deeper understanding of the specific types of interactions driving the binding process. Electrostatic interactions (negative values) influence the optimal binding orientation of the ligand within the receptor’s active site, leading to conformational changes in both the ligand and the receptor, thereby impacting the overall fit and stability of the complex. They also influence the solvent effects, charged residues, and specificity of binding, followed by structural rearrangements. Hydrogen bonds are formed when a hydrogen atom of the ligand’s functional group interacts with electronegative atoms, such as oxygen or nitrogen, in the receptor (amino acids). Their presence, number, and strength contribute to the stability and specificity of the ligand–receptor complex, aiding in the accurate orientation of the ligand, and facilitating optimal interactions with the receptor’s active site residues. Van der Waals energy quantifies the favorable interactions between hydrophobic portions of the ligand and receptor. These interactions contribute to the snug fitting of the ligand into the receptor’s binding pocket, optimizing binding affinity and specificity. Solvation energies account for the energetics of solvating molecules, and they help simulate the ligand–receptor interactions in a more realistic physiological context, forming interactions with the receptor. They contribute to the overall binding free energy and provide insights into the balance between hydrophobic and hydrophilic interactions. To summarize, the assessment of these values during docking simulations provides a comprehensive understanding of the ligand’s compatibility with the receptor’s binding site. The detailed interactions of the top ten ligands are presented in Table 4 and Figure S1a–j

1. a_617: Cobicistat–MbtA complex (Figure S1a)

Cobicistat is a Cytochrome P450 3A inhibitor used as a pharmacokinetic enhancer in combination with certain HIV-1 protease inhibitors (PIs) to improve their effectiveness. This showed the highest binding energy of -16.69 kcal/mol, which signifies its effective binding in the active site of MbtA. Three H-bonds were made (Asn258, Thr462, and Arg451): (i) the OH^4^ of the carbonyl oxygen of a_617 to the nitrogen of Asn285 (OH^4^_Carbonyl oxygen_ − NH_Asn258_ = 2.80 Å); (ii) the N^7^H of the thiazole ring of a_617 to the nitrogen of Thr462 (N^7^H_Thiazole Ring_ − NH_Thr462_ = 3.06 Å) and the O^1^H of the carbonyl oxygen of a_617 to the oxygen (OG1) of Thr462 (O^1^H_Carbonyl oxygen_ − OH_Thr462_ = 2.98 Å); and (iii) the N^4^H of the thiazole ring of a_617 to the nitrogen^2^ (guanidine group) of Arg451 (N^4^H_Thiazole Ring_ − NH^2^_Arg451_ = 3.15 Å), the O^2^H of the carbonyl oxygen of a_617 to the nitrogen^2^ (guanidine group) of Arg451 (O^2^H_Carbonyl oxygen_ − NH^2^_Arg451_ = 2.48 Å), and the O^3^ of the morpholine ring of a_617 to the nitrogen^1^ (guanidine group) of Arg451 (O^3^_Morpholine ring_ − NH^1^_Arg451_ = 3.10 Å).

2. a_391: Saquinavir–MbtA complex (Figure S1b)

Saquinavir, an inhibitor of HIV protease, exhibited a binding energy of -16.33 kcal/mol. It established six H-bonds with active site residues (Asn258, Thr462, Arg451, Gly460, Ala356, and Phe259): (i) the carbonyl oxygen of N-tert-butylformamide (a_391) to the α-amino group (NH) of Asn258 (O_N-tert-butylformamide_ − NH_Asn258_ = 2.87 Å); (ii) the hydroxyl oxygen (attached to octahydroisoquinolin) of a_391 to the oxygen of Thr462 (O^2^H_Octahydroisoquinolin_ − OH_Thr462_ = 2.81 Å); (iii) the N of the quinoline ring of a_391 to the nitrogen^2^ (guanidino group) of Arg451 (N_Quinoline ring_ − NH^2^_Arg451_ = 3.21 Å) and the oxygen of the oxopropyl-quinoline of a_617 to the nitrogen^1^ (guanidino group) of Arg451 (O_Oxopropyl-quinoline_ − NH^1^_Arg451_ = 3.23 Å); (iv) the hydroxyl oxygen (attached to octahydroisoquinolin) of a_391 to the Oxygen of Gly460 (O^2^H_Octahydroisoquinolin_ − O_NH2-CH2-COOH (Gly460)_ = 3.05 Å); (v) the oxygen attached to quinoline-2-carboxamide (a_391) to the amino group (NH) of Ala356 (O_Quinoline-2-carboxamide_ − NH_Ala356_ = 2.92 Å); and (vi) the carbonyl oxygen of N-tert-butylformamide (a_617) to the amino group (NH) of Phe259 (O^1^_N-tert-butylformamide_ − NH_Phe259_ = 3.07 Å).

3. a_821: Carfilzomib–MbtA complex (Figure S1c)

Carfilzomib, acting as a proteasome inhibitor, exhibited a binding energy of −16.08 kcal/mol with three hydrogen bonds (Gly330, Thr462, and Arg451): (i) the N^1^H of N-methylacetamide in a_821 to the OH group of Gly330 (NH_N-methylacetamide_ − OH_Gly330_ = 2.95 Å); (ii) the oxygen of formamido-N-methylacetamide in a_821 to the side chain hydroxyl group of Thr462 (O^5^_Formamido-N-methylacetamide_ − OH_Thr462_ = 3.21 Å); and (iii) the oxygen^2^ of N-methylacetamide of a_821 to the nitrogen^2^ (guanidino group) of Arg451 (O^2^_N-methylacetamide_ − NH^2^_Arg451_ = 2.76 Å)

4. a_827: Candesartan–MbtA complex (Figure S1d)

Candesartan, an angiotensin II receptor antagonist, demonstrated a binding energy of −15.82 kcal/mol. It established five H-bonds with active site residues (Asn258, Arg451, Asp436, Gly354, and His257): (i) the oxygen of the cyclohexyl hydrogen carbonate part in a_827 to the NH of side chain carboxamide of Asn258 (O^4^_Cyclohexyl hydrogen carbonate_ − NH_Asn258_ = 2.96 Å) and the carbonyl oxygen (O^2^) in a_827 to the NH of the α-amino group of Asn258 (O^2^ − NH_Asn258_ = 2.82 Å); (ii) the N^6^ of the tetrazole ring of a_827 to the nitrogen^1^ (guanidino group) of Arg451 (N^6^_Tetrazole ring_ − NH^1^_Arg451_ = 3.06 Å) and to the nitrogen^2^ (guanidino group) of Arg451 (N^6^_Tetrazole ring_ − NH^2^_Arg451_ = 3.16 Å); (iii) the N^5^ of the tetrazole ring of a_827 to the acidic side chain (CH_2_COOH) of Asp436 (N^5^_Tetrazole ring_ − OH_Asp436_ = 2.72 Å); (iv) the N^1^ of dihydro-1H-imidazole of a_827 to the NH of Gly354 (N^1^_Dihydro-1H-imidazole_ − NH_Gly354_ = 3.01 Å); and (v) the oxygen of cyclohexanol in a_827 to the NH of the imidazole side chain of His257 (O^6^_Cyclohexanol_ − NH_His257_ = 2.87 Å).

5. a_85: Ritonavir–MbtA complex (Figure S1e)

Ritonavir, an HIV protease inhibitor, displayed a binding energy of -14.84 kcal/mol, engaging in four hydrogen bonds with the residues Ala356, Val212, Gly354, and His257: (i) the carbonyl oxygen of N-phenethyl acetamide (a_85) to the amino group (NH) of Ala356 (O_N-phenethyl acetamide_ − NH_Ala356_ = 2.88 Å); (ii) the oxygen^5^ in a_85 to the carbonyl oxygen of the α-carboxylic acid group of Val212 (O^5^_a_827_ − O_α-carboxylic acid:Val212_ = 2.80 Å); (iii) the N^2^ of the azanecarboxamide of a_85 to the carbonyl oxygen of Gly354 (N^1^_Azanecarboxamide_ − O_Gly354_ = 2.96 Å); (iv) and the oxygen^5^ in a_85 to the NH of imidazole side chain of His257 (O^5^_a_827_ − NH_His257_ = 3.08 Å).

6. a_472: Lopinavir–MbtA complex (Figure S1f)

Lopinavir, an HIV protease inhibitor employed in the treatment of HIV infection, exhibited a binding energy of −14.92 kcal/mol and formed four hydrogen bonds with the active site residues Gly354, Gly460, Thr462, and Arg451: (i) the N^1^H of N-ethylpropionamide of a_617 to the carbonyl oxygen of Gly354 (N^1^_HN-ethylpropionamide_ − O_Gly354_ = 2.75 Å); (ii) the hydroxyl oxygen^3^ (attached to N-(2-hydroxypropyl)acetamide) of a_472 to the oxygen of Gly460 (O^3^H_N-(2-hydroxypropyl)acetamide_ − O_NH2-CH2-COOH (Gly460)_ = 2.92 Å); (iii) the hydroxyl oxygen^3^ (attached to N-(2-hydroxypropyl)acetamide) of a_472 to the side chain hydroxyl group of Thr462 (O^3^H_N-(2-hydroxypropyl)acetamide_ − OH_Thr462_ = 3.21 Å) and the NH of the tetrahydro-pyrimidin-2-one of a_472 to the hydroxyl of the carboxyl group of Thr462 (NH_Tetrahydro-pyrimidin-2-one_ − OH_Thr462_ = 3.16 Å); and (iv) the carbonyl oxygen of N-methylacetamide of a_472 to the nitrogen^2^ (guanidino group) of Arg451 (O_N-methylacetamide_ − NH^2^_Arg451_ = 2.86 Å),

7. a_1276: Indinavir–MbtA complex (Figure S1g)

Indinavir, an integral component of highly active antiretroviral therapy for treating HIV/AIDS, exhibited a strong binding affinity of −14.80 kcal/mol and displayed two hydrogen bonds with the active site residues Gly354 and Arg451: (i) the NH of piperazine-2-carboxamide of a_1276 to the carbonyl oxygen of Gly354 (NH_Piperazine-2-carboxamide_ − O_Gly354_ = 2.66 Å) and (ii) the carbonyl oxygen of piperazine-2-carboxamide of a_1276 to the nitrogen^2^ (guanidino group) of Arg451 (O_Piperazine-2-carboxamide_ − NH^2^_Arg451_ = 2.63 Å) and the nitrogen of pyridine of a_1276 to the nitrogen^1^ (guanidino group) of Arg451 (O_Pyridine_ − NH^1^_Arg451_ = 2.93 Å).

8. a_1338: Venetoclax–MbtA complex (Figure S1h)

Venetoclax, a B-cell lymphoma-2 (BCL-2) inhibitor known for its anti-apoptotic role, exhibited robust binding to MbtA with a binding energy of −14.68 kcal/mol and established three hydrogen bonds with the residues Val352, Thr462, and Phe259: (i) the nitrogen of the benzenesulfonamide of a_1338 to the hydroxyl group of the α-carboxylic acid group of Val352 (N_Benzenesulfonamide_ − OH_Val352_ = 3.17 Å); (ii) the oxygen of tetrahydro-2H-pyran of a_1338 to the side chain hydroxyl group of Thr462 (O_Tetrahydro-2H-pyran_ − OH_Thr462_ = 2.83 Å); and (iii) the carbonyl oxygen of nitrobenzene of a_1338 to the side chain amino group (NH) of Phe259 (O^6^_Nitrobenzene_ − NH_Phe259_ = 3.07 Å).

9. a_797: Zafirlukast (Accolate)–MbtA complex (Figure S1i)

Zafirlukast, a leukotriene receptor antagonist, demonstrated binding to MbtA with a binding energy of −14.50 kcal/mol and formed three hydrogen bonds with the active site residues Asn258, His257, and Arg451: (i) the carbonyl oxygen of cyclopentyl methylcarbamate (a_797) to the α-amino group (NH) of Asn258 (O_Cyclopentyl methylcarbamate_ − NH_Asn258_ = 3.07 Å); (ii) the carbonyl oxygen of cyclopentyl methylcarbamate (a_797) to the NH of the imidazole side chain of His257 (O_Cyclopentyl methylcarbamate_ − NH_His257_ = 3.04 Å); and (iii) the carbonyl oxygen of benzenesulfonamide of a_797 to the NH (guanidino group) of Arg451 (O^6^_Benzenesulfonamide_ − NH_Arg451_ = 2.87 Å) and the hydroxyl of benzimidic acid of a_797 to the nitrogen^2^ (guanidino group) of Arg451 (O_Benzimidic acid_ − NH^2^_Arg451_ = 2.88 Å).

10. a_1388: Neratinib–MbtA complex (Figure S1j)

Neratinib, recognized for its anticancer properties as a tyrosine kinase inhibitor, displayed a binding energy of −15.20 kcal/mol while establishing three hydrogen bonds with the active site residues His257, Phe259, and Thr462: (i) the oxygen attached to the dihydroquinoline of a_1388 to the NH of the imidazole side chain of His257 (O_Dihydroquinoline_ − NH_His257_ = 2.88 Å); (ii) carbonyl oxygen of the N-methylacrylamide of a_1388 to the side chain amino group (NH) of Phe259 (O^2^_N-methylacrylamide_ − NH_Phe259_ = 2.94 Å); and (iii) the bridging nitrogen (N3) of a_1388 to the side chain hydroxyl group of Thr462 (N^3^_Linker bridge_ − OH_Thr462_ = 2.94 Å).

The active site residues (amino acids) Asn258, Thr462, Arg451, Gly460, Ala356, Phe259, Gly330, Asp436, Gly354, His257, Val212, and Val352 crucially show a significant contribution towards the binding of ligands in the active site of MbtA by forming hydrogen bonds with external ligands, particularly in the context of tuberculosis (TB) and mycobactin biosynthesis [58]. These interactions contribute to substrate recognition, binding, and catalysis within the active site. Asn258 is likely involved in forming hydrogen bonds with ligands, aiding in their precise orientation and stabilization within the active site. Thr462 and Arg451 form multiple hydrogen bonds to anchor ligands and stabilize their binding conformation within the active site. The presence of Gly460 can influence the conformation and flexibility of nearby residues, potentially affecting ligand interactions. Ala356 may contribute to the overall structural stability of the active site and provide a hydrophobic environment for ligand binding. Phe259 and His257 can form π-π interactions or other types of hydrogen bonds with ligands, aiding in their recognition, stabilization, and binding specificity. Gly330, being similar to Gly460, may influence the flexibility of nearby residues and contribute to the overall dynamics of ligand binding. Asp436 can form hydrogen bonds with ligands and assist in positioning them for catalysis or recognition. Gly354, as with other glycine residues, can impact the overall flexibility of active site region, influencing ligand interactions. Val212 and Val352 potentially create a hydrophobic environment within the active site, aiding in ligand binding through hydrogen bonds and hydrophobic interactions while also assisting in the proper positioning of ligands. All other active site residues play a role in facilitating hydrophobic interactions by protein folding, contributing significantly to the stability and biological activity of proteins. They enable proteins to minimize their surface area, thereby reducing unfavorable interactions with water. Hence, in the context of MbtA, the formation of hydrogen bonds and hydrophobic residues between these amino acids and external ligands contributes to the specificity and efficiency of substrate/ligand binding and catalytic processes.

### 3.2. Molecular Dynamics Simulation Study

To gain a more profound insight into the binding mechanism and validate the ligand–protein complexes, we conducted 300 ns molecular dynamics (MD) simulations to analyze the biophysical interactions between the docked complexes (seven in total). In addition, a 300 ns simulation of the apo-protein was conducted. This endeavor aims to facilitate the drawing of scientific conclusions regarding the biophysical interactions influencing protein stability and to elucidate any ligand-induced conformational changes. These selections encompassed a wide range of compound classes of interest, including protease inhibitors utilized in HIV therapy, CYP inhibitors, ACE inhibitors, and anticancer agents. These identified compounds have the potential to act as dual inhibitors, exhibiting both binding affinity to and inhibition of MbtA. The evaluation included root mean square deviation (RMSD), root mean square fluctuation (RMSF), and the percentage of interactions between the ligands and protein atoms. The retention of interactions observed during docking was also verified over the 300 ns MD simulation. Conformational changes were measured using RMSD values, where the initial frame served as the reference backbone, and the deviation at the end was compared to the starting point. The system achieved stabilization and equilibration when the RMSD values ranged from 0.1 to 1.5 Å. Lower complex stability was associated with more significant fluctuations. This analysis allowed us to ascertain the reliability of the ligand–protein interactions and provided insights into the dynamic behavior of the complexes during the simulation.

**Figure 6:**
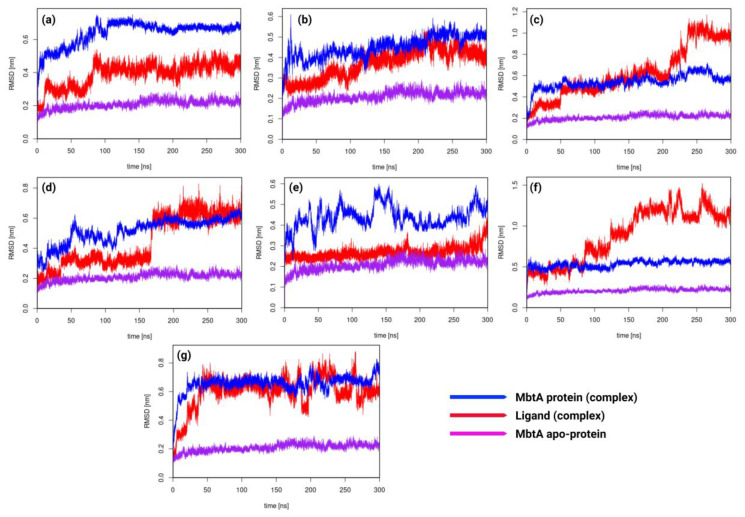
Root mean square deviation (RMSD) plot of MbtA protein (blue) with ligands (red) (**a**) a_617: Cobicistat, (**b**) a_391: Saquinavir, (**c**) a_821: Carfilzomib, (**d**) a_827: Candesartan, (**e**) a_85: Ritonavir, (**f**) a_472: Lopinavir, and (**g**) a_1276: Indinavir and apo-protein (purple).

*RMSD analysis:* The apo-MbtA protein demonstrated remarkable stability throughout the simulation, maintaining a consistent root mean square deviation (RMSD) within the range of 0.15 to 0.25 nm. It is evident that upon the binding of any ligand to the apo-protein, deviations are expected. However, these deviations should be minimal to ensure that the stability of the protein is not compromised. In complex a_617-MbtA, initially, the RMSD was volatile till 100 ns, but then, it was stable throughout the simulation with RMSDs of 0.6 nm (protein) and 0.4 nm (ligand). For complex a_391-MbtA, the complex was stable throughout the simulation with constant RMSDs of 0.45 nm (protein) and 0.35 nm (ligand). There were mild fluctuations in the initial frames (0–30 ns), but eventually, the complex stabilized around fixed values. For complex a_821-MbtA, the complex was stable between 50 and 200 ns at a fixed RMSD of 0.5nm. Fluctuations were observed initially in both protein and ligand, but after 200 ns, the ligand displayed fluctuations with RMSDs in the range of 0.7–1.1 nm. For complex a_827-MbtA, the protein fluctuated significantly for the first 110 ns. However, it then attained stability and had an RMSD of 0.5 nm. The ligand was stable throughout the course of the simulation. However, it displayed stability at two RMSD values, 0–160 ns at 0.25 nm and 160–300 ns at 0.6 nm. Overall, the complex was stable from 160 to 300 ns at an RMSD of 0.6 nm. For complex a_85-MbtA, the ligand was stable throughout the simulation (0–300 ns) at a constant RMSD of 0.25 nm, while the protein fluctuated a lot initially (0–160 ns: 0.3–0.55 nm) and towards the end of the simulation (280–300 ns: 0.45–0.55 nm), and eventually, in mid-segment, it gained stability (160–280 ns) at an RMSD of 0.4 nm. In total, the complex was stable from 160 to 280 ns. For complex a_472-MbtA, it was very surprising to observe that the protein was stable throughout the course of the simulation (0–300 ns) at a constant RMSD of 0.5 nm. The ligand was stable initially (0–90 ns) at an RMSD of 0.5, but eventually, it had major fluctuations throughout the rest of the simulation (90–300 ns). Overall, the complex was stable from 0 to 90 ns at an RMSD of 0.5, and for complex a_1276-MbtA, there were initial fluctuations in RMSD for both protein and ligand, but eventually, from 50 to 160 ns, both the protein and ligand had a constant RMSD of 0.6nm, after which (160–300 ns) there were minor fluctuations, but the RMSD window was large (protein: 0.45–0.8 and ligand: 0.4–0.8 nm). The identified trends indicate sustained stability across all protein–ligand complexes and apo-proteins during the simulation, punctuated by minor fluctuations, as demonstrated in Figure 6. Among these, PLC (a), (b), (c), (f), and (g) displayed remarkable stability throughout the simulation duration. Meanwhile, PLC (d) and (e) exhibited intermittent fluctuations; nevertheless, they maintained a commendable level of overall stability. Despite a slight increase in the RMSD values for the MbtA protein with bound ligands, the consistency observed suggests the stability of the PLCs when compared to the RMSD of apo-protein.

**Figure 7:**
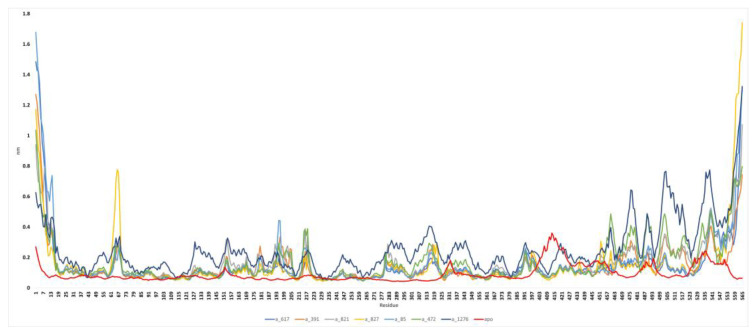
Root mean square fluctuation (RMSF) plot of MbtA protein with ligands (a) a_617: Cobicistat (blue), (b) a_391: Saquinavir (orange), (c) a_821: Carfilzomib (grey), (d) a_827: Candesartan (yellow), (e) a_85: Ritonavir (blue), (f) a_472: Lopinavir (green), and (g) a_1276: Indinavir (purple) and apo-protein (red).

*RMSF analysis:* RMSF is a measure of the fluctuation of atomic positions in a biomolecular system over the course of a simulation and is determined by the root mean square deviation of each atom’s position from its average position over the course of a simulation. The peaks visible in Figure 7 correspond to areas of the protein that exhibited the highest levels of fluctuation throughout the simulation. Based on our experience, the N- and C-terminal tails of the protein tend to be the most dynamic and variable regions. Distinct variations were noted in the RMSF plots for C-alpha residues in the ranges of 60–70, 180–220, 280–320, and 450–550 across all seven complexes. These observed variations are likely indicative of the spatial adjustments occurring in the active site residues upon binding of the ligands. In the case of complex a_827-MbtA, noticeable fluctuations were evident, as depicted by a distinct peak in the 60–70 residue range due to their flexibility. For complex a_472-MbtA, a substantial number of variations were observed in residues 450–550. Nevertheless, it is worth noting that all complexes exhibited satisfactory RMSF values, indicating their overall stability. It is also evident that specific active site residues exhibit a degree of flexibility in the presence of ligand-bound complexes. In the case of the apo-protein, initially, there were no fluctuations; however, minor fluctuations were observed in residues in the ranges of 403–427 and 529–553. This suggests that the formation of complexes with inhibitors contributes to the stabilization of the active site.

**Figure 8:**
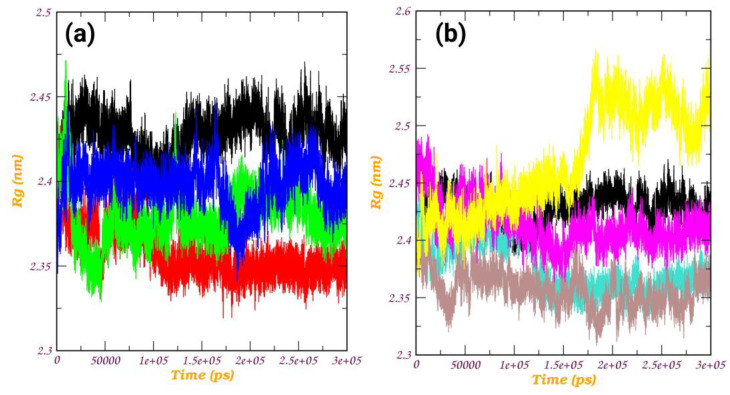
Radius of gyration (Rg) plot of (**a**) MbtA protein with ligands (i) a_617: Cobicistat (red), (ii) a_391: Saquinavir (green), and (iii) a_821: Carfilzomib (blue) and (v) MbtA apo-protein (black); (**b**) MbtA protein with ligands (i) a_827: Candesartan (turquoise), (ii) a_85: Ritonavir (brown), (iii) a_472: Lopinavir (magenta), and (iv) a_1276: Indinavir (yellow) and (v) MbtA apo-protein (black)

*Rg analysis:* The radius of gyration (Rg) plot is a graphical representation that illustrates the compactness or size of a molecule during simulation, as shown in Figure 8. The radius of gyration measures the average distance of each atom in a molecule from its center of mass. For a protein, a lower radius of gyration indicates a more compact and folded structure, while a higher value suggests a more extended or unfolded structure. In our investigation, all protein–ligand complexes, with the exception of the a_1276-MbtA complex, exhibited an initial expansion in the size of the protein–ligand assembly. This phenomenon pointed to conformational changes occurring during the simulation, which were subsequently followed by a reduction and stabilization phase around 50 ns. Beyond this point, intermittent spikes were evident, suggesting a certain degree of structural stability and the emergence of enduring interactions between the protein and the ligand. However, the a_1276-MbtA complex displayed commendable stability initially, maintaining this state until approximately 60 ns. Following this period, there was a sharp rise in spikes and an increase in the size of the protein–ligand assembly after 150 ns, indicative of a substantial conformational alteration. Despite this, the complex managed to attain stability again, albeit at a higher value. The oscillations seen in the Rg plot signify instances of unfolding or alterations in the conformation, while the plateaus indicate periods of steady structural arrangements. Notably, the protein–ligand complex initiates the simulation with observable conformational changes, as evidenced by the initial expansion in size. Subsequently, the complex experiences stabilization, evident through a decrease in and steadying of the Rg values, interspersed with episodes of fluctuations. This pattern strongly suggests the development of enduring interactions between the protein and the ligand, progressively solidifying as the simulation unfolds. These observations align with the enduring interactions observed in the apo-protein, indicating consistency in the structural behavior of the specified residues.

**Figure 9:**
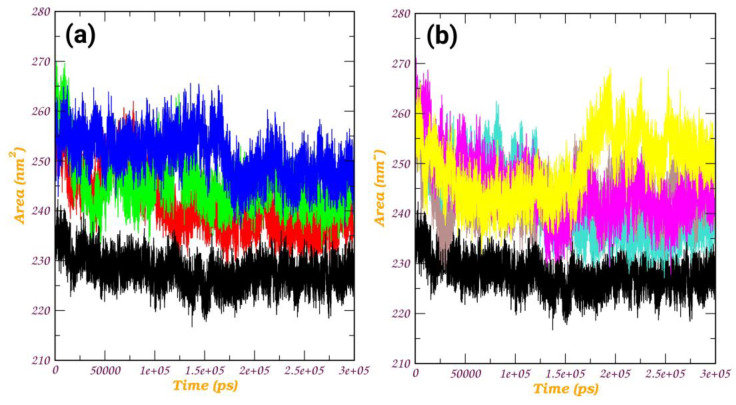
Solvent-accessible surface area (SASA) plot of (**a**) MbtA protein with ligands (i) a_617: Cobicistat (red), (ii) a_391: Saquinavir (green), and (iii) a_821: Carfilzomib (blue) and (v) MbtA apo-protein (black); (**b**) MbtA protein with ligands (i) a_827: Candesartan (turquoise), (ii) a_85: Ritonavir (brown), (iii) a_472: Lopinavir (magenta), and (iv) a_1276: Indinavir (yellow) and (v) MbtA apo-protein (black).

***SASA Analysis:*** SASA quantifies the surface area of a molecule that is accessible to solvent molecules, providing valuable insights into the molecule’s exposure to its surrounding environment. In the case of the apo-protein, there was a gradual exposure of certain residues to the solvent over the course of the simulation, showing an upward trend. In our study, apart from the a_1276-MbtA complex, all other complexes exhibited a comparable exposure pattern. They demonstrated an initial rise between 0 and 25 ns, followed by a consistently stable graph with minimal spikes and fluctuations throughout the entire simulation period. The general upward trend in SASA across the a_1276-MbtA complex suggests that the protein–ligand interactions gradually expose themselves to the solvent over the course of the simulation, while this was constant for other ligands as they converged around fixed values. The rise in SASA could potentially signify unfolding or conformational changes, whereas a decrease might imply the adoption of more compact and structurally stable conformations. These observations align with those of the apo-protein. This observation attests to the stability of all the protein–ligand complexes over the simulation time, as shown in Figure 9.

**Time Frame Analysis with a focus on H-bond and active site residues:** Frame analysis in molecular dynamics refers to the systematic examination and evaluation of the molecular structure and behavior at specific time intervals or frames during a simulation. It plays a crucial role in understanding the dynamics, stability, and interactions of molecules over time. This total simulation period is divided into discrete time frames or frames, where each frame represents a specific point in time. Upon analysis of the highest-ranking molecules from Table 3, time frame analysis was conducted by extracting a snapshot of the frame from the region of most excellent stability, as indicated by the RMSD (root mean square deviation) graph. The *gmx_trjconv* module was employed to take a snapshot. It is evident that during the simulation, this highly stable frame represents the interactions that are most likely to remain constant. These stable interactions are crucial for the effective binding of the ligand to the receptor. The detailed analysis is as follows: (a) The a_617-MbtA complex made one H-bond: the nitrogen of the thiazole of a_617 to the α-amino group (NH) of Gly214 (N^6^_Thiazole_ − NH_Gly214_ = 3.07 Å). (b) The a_391-MbtA complex made four H-bonds: (i) the carbonyl oxygen attached to quinoline-2-carboxamide to the α-amino group (NH) of Gly214 (O^2^_Quinoline-2-carboxamide_ − NH_Gly215_ = 3.02 Å); (ii) the carbonyl oxygen attached to quinoline-2-carboxamide to the α-amino group (NH) of Gly215 (O^2^_Quinoline-2-carboxamide_ − NH_Gly215_ = 3.16 Å); (iii) the carbonyl oxygen (attached to octahydroisoquinolin) of a_391 to the NH of Thr462 (O^1^_Octahydroisoquinolin_ − NH_Thr462_ = 3.07 Å) and the N^3^H of a_391 to the carbonyl oxygen of Thr462 (N^3^H_a_391_ − O_Thr462_ = 3.02 Å) (this interaction was conserved as in docking); (iv) the carbonyl oxygen (attached to octahydroisoquinolin) of a_391 to the OH of carboxylic acid of Glu461 (O^1^_Octahydroisoquinolin_ − OH_Glu461_ = 2.81 Å) and the N^1^H of a_391 to the OH of carboxylic acid of Glu461 (N^1^H_a_391_ − OH_Glu461_ = 2.86 Å). (c) The a_821-MbtA complex made two H-bonds: (i) the NH of N-methylacetamide in a_821 to the OH group of Gly330 (NH_N-methylacetamide_ − OH_Gly330_ = 2.79 Å), and (ii) the NH of N-methyl-2-morpholinoacetamide in a_821 to the OH group of Gly460 (NH_N-methyl-2-morpholinoacetamide_ − OH_Gly460_ = 2.93 Å). As an observation, it was seen in the top three molecules that the majority of the hydrogen bonds were established with glycine residue. The presence of glycine can influence the conformation and flexibility of nearby residues, potentially affecting ligand interactions due to its small and structurally flexible nature. It also facilitates specific interactions with ligands. However, the specific role of glycine residues in the active site of MbtA can vary depending on their positions within the protein’s primary structure. For (d) the a_827-MbtA complex, there were two H-bonds: (i) the N^4^ of the tetrazole ring of a_827 to the acidic side chain (CH_2_COOH) of Asp436 (N^4^_Tetrazole ring_ − OH_Asp436_ = 2.53 Å), and (ii) the nitrogen of imidazole in a_827 to the NH of the imidazole side chain of His257 (N_Imidazole_ − NH_His257_ = 2.84 Å). The a_85-MbtA complex displayed two H-bonds: (i) the carbonyl oxygen of N-phenethyl acetamide (a_85) to the amino group (NH) of Ala356 (O_N-phenethyl acetamide_ − NH_Ala356_ = 2.66 Å), and (ii) the carbonyl oxygen of a_85 to the amino group (NH) of Phe259 (O^4^_a_391_ − NH_Phe259_ = 3.22 Å). Complex a_472-MbtA made one H-bond: the NH of tetrahydro-pyrimidin-2-one of a_472 to the hydroxyl of the carboxyl group of Thr462 (NH_Tetrahydro-pyrimidin-2-one_ − OH_Thr462_ = 2.86 Å). The a_1276-MbtA complex showed no H-bonding. The majority of interactions remained consistent across all four of these protein–ligand complexes. Figure S2 displays the interactions made by the ligands in complex with MbtA for the most stable frame during MD simulation, as evidenced by the RMSD graph. The research pertaining to the exploration of the crystal structure of MbtA as an antitubercular target is at a very initial stage and calls for much more evidence-based literature. In summary, the frame analysis of our top-scoring PLCs provides a systematic examination of molecular properties and interactions at discrete time intervals for the most stable frame.

**Comparison between the conformations explored by the apo-protein with respect to the protein–ligand complexes:** This analysis is of significant importance in elucidating the structural dynamics and potential functional implications to identify any ligand-induced conformational changes, and provides insights into the stability and behavior of the protein in different states. In this study, a specific frame was extracted from PLC taken from the most stable region, as indicated by the RMSD and RMSF plots. These frames were saved as “*frame.pdb*.” Similarly, a stable frame was also extracted from the apo-protein using the *traj_conv* command and saved as “*apo_frame.pdb*.” Subsequently, these frames were superimposed, and the RMSD was calculated. Additionally, 2D maps of residue–residue distances, including the (a) distance and standard deviation and (b) difference, were computed, and corresponding graphs were generated for analysis.

For the a_617-MbtA complex, the RMSD between 359 pruned atom pairs is 1.023 Å (across all 535 pairs: 14.563 Å); for the a_391-MbtA complex, the RMSD between 358 pruned atom pairs is 0.937 Å (across all 535 pairs: 14.520); for the a_821-MbtA complex, the RMSD between 362 pruned atom pairs is 1.041 Å (across all 535 pairs: 14.131); for the a_827-MbtA complex, the RMSD between 369 pruned atom pairs is 1.059 Å (across all 535 pairs: 14.650); for the a_85-MbtA complex, the RMSD between 345 pruned atom pairs is 1.001 Å (across all 535 pairs: 13.204); for the a_472-MbtA complex, the RMSD between 348 pruned atom pairs is 1.107 Å (across all 535 pairs: 10.987); and for the a_1276-MbtA complex, the RMSD between 376 pruned atom pairs is 0.950 Å (across all 535 pairs: 13.315).

The observed RMSD values for various complexes of a_617-MbtA, a_391-MbtA, a_821-MbtA, a_827-MbtA, a_85-MbtA, a_472-MbtA, and a_1276-MbtA reveal the extent of structural deviation between the pruned atom pairs. These values indicate the degree of deviation in atomic positions for each complex, offering a nuanced understanding of how different ligands influence the structural stability of the MbtA protein. Notably, each complex exhibits a distinct RMSD value, indicating the variability in the conformational changes within the specified regions. For instance, the a_472-MbtA complex displays a relatively higher RMSD of 1.107 Å, suggesting greater structural divergence in comparison to other complexes. The assessment across all 535 pairs provides a broader perspective, showing the overall fluctuation in atomic positions across these complexes. These findings contribute valuable insights into the dynamic behavior and stability of the respective protein–ligand complexes. The observed very minor fluctuations in the RMSD values upon ligand binding, in comparison to the apo-protein, suggest remarkable stability in the protein–ligand complexes. This stability is indicative of a robust and well-maintained structural integrity in the presence of ligands. These findings lend support to the hypothesis that the interactions between the protein and ligands contribute to the overall stability of the complexes. The consistency in structural behavior further underscores the reliability and endurance of the protein–ligand interactions, reinforcing the significance of these complexes in maintaining stable conformations. The detailed graphical analysis is presented in Figure S3.

## 4. Principal Component Analysis (PCA)

**Figure 10:**
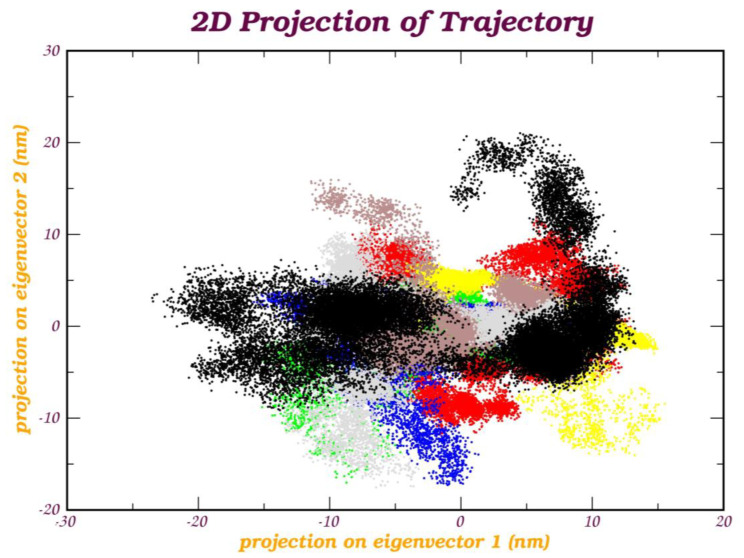
Principal component analysis of protein–ligand complexes: the collective motion of (a) a_617: Cobicistat (yellow), (b) a_391: Saquinavir (green), (c) a_821: Carfilzomib (brown), (d) a_827: Candesartan (grey), (e) a_85: Ritonavir (red), (f) a_472: Lopinavir (blue), and (g) a_1276: Indinavir (black) with MbtA using projections of MD trajectories on two eigenvectors corresponding to the first two principal components.

Principal component analysis (PCA) investigated the primary modes of motion within the simulation trajectory. To comprehend the primary structural variations elucidated by each MbtA-ligand complex, we generated PCA graphs. The collective motion represented by the first two principal components (PCs) and 2D projections of PC1 and PC2 were plotted for all the protein–ligand complexes (PLCs), as depicted in Figure 10. As an observation, it is seen that the complexes a_85-MbtA, a_821-MbtA, and a_1276-MbtA express compact clusters in the conformational spaces that range from −10 to 10; for the latter two PLCs, there are slight scatterings across the configurational space (Figure 10e,c,g). In the MD trajectory of a_617-MbtA and a_391-MbtA, complexes PC1 and PC2 (top two modes) show a varied constant distribution across the configurational space with little variation in the conformational space, and are widely grouped in the range of −15 to 5. (Figure 10a,b). In the case of the a_827-MbtA and a_472-MbtA complexes, the graph displays a varied distribution across the configurational space with slightly more variation, as evident from the widely grouped data in the range of −5 to 10 (Figure 10d,f).

In these plots (Figure S4), distinct color transitions are evident at various points, signifying shifts between different conformations induced by ligand binding. Each data point represents an individual frame within the trajectory. Throughout the 300 ns simulation, the systems generally maintained compactness, with minor deviations observed for all PLCs. These fluctuations could be attributed to temporary disruptions in hydrogen bonds or the accommodation of ligands within the active site. These analyses underscore the considerable flexibility of the protein structure when interacting with the seven hit compounds during the initial simulation phases, with a gradual reduction in flexibility as the simulation progressed. Furthermore, the contribution percentage in eigenmodes consistently decreased, indicating a tendency towards local structural stabilization in each complex. These motions primarily stemmed from the interactions of the docked compounds within the active site of the protein, reinforcing the formation of robust complexes for each protein–ligand pair.

The root mean square fluctuation (RMSF) nm of eigenvectors—the backbone in PCA of molecular dynamics trajectories—refers to the measure of atomic fluctuations or deviations from their average positions within a protein over a period of time during a simulation, thereby providing insights into the flexibility and mobility of specific regions or residues within the structure. Herein, as an observation, it was seen that vector 1 displayed fluctuations in the atom number range of 200–250, and all other vectors displayed significant fluctuations in the atom range of 1250–1750, except vector 3, for all PLCs. This information provides insights into the relative stability of different parts of the molecule throughout the simulation. It also assists in understanding structural dynamics and may offer valuable clues about functional or binding sites. The minimal fluctuations observed indicate that all seven PLCs maintain stability within the active site of MbtA, suggesting their potential contribution to effective inhibition. A graphical representation of RMSF is presented in Figures S5 and S6

In summary, PCA analysis revealed that the interconnected motion within the MbtA conformation reflects both the rigidity and significant variations induced at the active site as a consequence of ligand binding throughout the simulation. These analyses strongly indicate the stability of the top seven selected compounds within the MbtA active site, which, in turn, hinders the essential motions required for enzymatic activity. Multiple factors, including RMSD, RMSF, Rg, protein–ligand interactions, PCA studies, and numerous molecular docking scores, substantiate this inhibition.

## 5. Discussion

Tackling TB is crucial for improving public health, ensuring global health security, and advancing toward a world where infectious diseases are less of a threat to human well-being. Mycobactin biosynthesis machinery is conserved among all species of mycobacteria and presents a novel target for antitubercular drug development. Our primary focus lies on MbtA due to its pivotal role in catalyzing the initial step of mycobactin biosynthesis and its critical role in bacterial growth and virulence. Moreover, the modulation of efflux pumps could also help tackle the emergence of AMR in TB. Hence, to rediscover a drug with alternative therapeutic applications as an MbtA inhibitor, we conducted virtual screening using the FDA-approved drug library against the active pocket of the MbtA receptor. Our virtual screening yielded promising results, indicating strong binding affinities. Based on the calculated binding energies, we selected the top ten molecules for further investigation. Surprisingly, the top hits encompassed a diverse range of significant pharmacological classes: four molecules (a_391: Saquinavir, a_85: Ritonavir, a_472: Lopinavir, and a_1276: Indinavir) are well-known protease inhibitors widely utilized in antiviral therapies, three molecules are anticancer agents (a_821: Carfilzomib, a_1338: Venetoclax, and a_1388: Neratinib), one functions as a CYP450 inhibitor (a_617: Cobicistat), one as an ACE inhibitor (a_827: Candesartan), and one as a leukotriene antagonist (a_797: Zafirlukast, or Accolate). These compounds exhibited docking scores ranging from −16.69 to −14.14 kcal/mol, indicative of strong and specific interactions with the target receptor. The interaction between the ligands and the amino acid residues within the active site of MbtA plays a critical role in substrate recognition, binding, and catalysis. Key active site amino acid residues, including Asn258, Thr462, Arg451, Gly460, Ala356, Phe259, Gly330, Asp436, Gly354, His257, Val212, and Val352, participate in hydrogen bonding and various other interactions with the ligands. These interactions anchor the ligands and stabilize their binding conformation within the active site, potentially influencing the conformation and flexibility of neighboring residues. To gain a deeper understanding of the binding characteristics, we conducted molecular dynamics simulations (300 ns) for the top seven molecules. Analysis of the root mean square deviation (RMSD) revealed the stability of all seven protein–ligand complexes (PLCs). The protein exhibited RMSD values within the range of 0.2–0.6 nm, while the ligands displayed RMSD values ranging from 0.2 to 1.5 nm, suggesting stable and consistent interactions within the complexes. The stability of the PLCs was confirmed through the comparison of protein and ligand RMSD graphs. These graphs exhibited consistent, smooth curves with minimal spikes for all PLCs, indicating their stable behavior throughout the simulation (average 100 ns). The analysis of root mean square fluctuation (RMSF) showed minimal atomic position fluctuations within the PLCs. However, distinct variations were observed in the RMSF plots for C-alpha residues, particularly in the ranges of 60–70, 180–220, 280–320, and 450–550, across all seven complexes. These variations likely signify spatial adjustments occurring within the active site residues upon ligand binding. Furthermore, it is evident that specific active site residues display a degree of flexibility when in the presence of ligand-bound complexes. This suggests that the formation of these complexes with inhibitors plays a role in stabilizing the active site. The analysis of the radius of gyration (Rg) for all PLCs revealed a notable pattern in the size of the protein–ligand assembly during the simulation. Initially, there was an expansion in the size, indicating conformational changes taking place. This phase was followed by a reduction and stabilization around the 50 ns mark. Beyond this point, intermittent spikes were observed, signifying a degree of structural stability and the emergence of enduring interactions between the protein and the ligand. The analysis of solvent-accessible surface area (SASA) revealed a consistent exposure pattern among all protein–ligand complexes (PLCs). Initially, there was an upward trend observed between 0 and 25 ns, which was succeeded by a persistently stable graph with minimal spikes and fluctuations throughout the entire simulation duration. The increase in SASA values during the initial phase may suggest unfolding or conformational alterations within the complexes. Conversely, a subsequent decrease could indicate the adoption of more compact and structurally stable conformations. This observation provides evidence of the stability maintained by all the protein–ligand complexes throughout the simulation period. The obtained results align with those of the apo-protein, indicating minimal ligand-induced conformational changes. This consistency underscores the stability of the protein structure, emphasizing the reliability of the observed trends across different conditions and providing confidence in the conclusions drawn from the study. To gain more insight into the binding modes and to identify structural changes in molecules or complexes, a particular time frame analysis (trajectory snapshot) was carried out for each PLCs from the most stable region of the RMSD plot. The results revealed that intermolecular interactions change over time, including atomic positions, velocities, energies, bond lengths, angles, dihedral angles, and solvent interactions, among others. This also highlights the system’s dynamics, stability, and equilibrium behavior (H-bond interaction) over time. The stability of the protein–ligand complexes (PLCs) could be attributed to the establishment of hydrogen bonds with specific residues. Notably, residues Gly214, Gly215, Glu461, Thr462, Gly460, Gly330, Asp436, His257, Phe259, Ala356, and Thr216 are involved in forming these hydrogen bonds, which likely play a significant role in ensuring stable binding mechanisms. To further validate these binding modalities, the extracted stable frames of each protein–ligand complex (PLC) were superimposed onto the stable frame of the apo-protein. The results revealed minimal root mean square deviation (RMSD), indicating that there were negligible ligand-induced conformational changes. This suggests a high level of stability in the protein–ligand complexes, affirming the robustness of their structural integrity. Furthermore, principal component analysis (PCA) substantiated the earlier findings, highlighting noteworthy motions and stabilities within the MbtA protein when bound to the top seven ligands. In summary, these seven molecules show promise as potential MbtA inhibitors due to their strong affinity for the active site and their potential to act as substrates for enzyme inhibition. Hence, this current study is solely based on computational approaches to assess the inhibitory interaction of FDA-reported molecules against MbtA.

## 6. Conclusions

Mycobacterial machinery constitutes an intricately complex process, demanding immediate attention for drug discovery. This urgency stems from the emergence of diverse resistant strains and the waning efficacy of current drugs. Taking into account the severity of this bacterial infestation along with antimicrobial resistance, there is a need to design, discover, and rediscover drugs that act on a novel target via a novel mechanism of action and that have broad-spectrum antibacterial activity. Our study focuses on targeting the critical dual enzyme system, MbtA-MbtB, which plays a vital role in the biosynthesis of iron-chelating mycobacterial siderophores. Inhibiting these enzymes with a drug would not only disrupt siderophore production machinery but also inhibit the operation of efflux pumps. Employing the concept of drug repurposing, also known as drug repositioning or reprofiling, we evaluated the FDA library of drugs against the MbtA protein. We, fortunately, ended up rediscovering seven hit molecules that were initially developed for different therapeutic indications as MbtA inhibitors. The majority of the revealed hits were identified as antivirals commonly used in the treatment of HIV/AIDS. This discovery presents an intriguing opportunity, suggesting that these hits, if proven effective in inhibiting MbtA in mycobactin machinery, could potentially serve as potent antitubercular agents. This dual functionality raises the prospect of addressing global challenges related to coinfections, particularly in HIV patients, who are more susceptible to such coinfections due to compromised immune systems. We employed the concepts of virtual screening and also provided a discussion on the binding modes and interactions between these drugs and MbtA, shedding light on the structural basis of their inhibitory potential. It is noteworthy that these molecules exhibit well-established ADMET profiles, further bolstering their suitability for repurposing. This detailed in silico study, incorporating molecular docking, molecular dynamics, and PCA analysis, sheds light on the significant role of in silico tools in repurposing FDA-approved drugs for TB therapy (MbtA inhibitor). PCA served to validate the consistency and reliability of our MD simulations. It identified and quantified the major collective motions within the PLC’s molecular system. This is crucial for understanding the essential dynamics, such as global conformational changes or specific protein motions, providing insights into the functional behavior of biomolecules. The utilization of computational methods provides valuable insights into the potential of existing drugs for new therapeutic applications, showcasing the versatility and efficiency of in silico approaches in drug discovery and development. This innovative utilization of drug repurposing not only underscores the adaptability of existing pharmaceuticals but also offers a practical avenue for effectively addressing the complexities of co-infection scenarios in today’s healthcare landscape.

In conclusion, our comprehensive in silico study, encompassing molecular docking, molecular dynamics, and PCA analysis, has provided valuable insights into the potential repurposing of FDA-approved drugs. The exploration of protein–ligand complexes (PLCs) revealed minimal ligand-induced conformational changes, consistent with the stability observed in the apo-protein. The fact that extracted stable frames of each PLC exhibited minimal RMSD when superimposed onto the stable frame of the apo-protein underscores the stability of these complexes. Overall, this study highlights the efficacy of in silico tools in drug repurposing efforts, providing a foundation for further experimental validation. These findings contribute to our understanding of potential therapeutic avenues and underscore the significance of computational approaches in accelerating drug discovery processes.

## 7. Future Scope

As previously mentioned, it was quite remarkable to discover that among the top 10 molecules, four of them are protease inhibitors widely used in antiviral therapies, particularly effective against HIV. This suggests the potential for these compounds to act as dual inhibitors in tackling cases of co-infection. The future direction of our research involves evaluating these molecules for their inhibitory activity against MbtA in both GAST and GAST-Fe media against *M. smegmatis* and *M. tuberculosis*. Additionally, we plan to conduct a Universal CAS siderophore inhibition assay in *M. smegmatis*. These assays represent a crucial step towards uncovering the dual inhibitory nature of these FDA-reported molecules. Having established safety and efficacy profiles, these drugs have the potential to become remarkable assets in the near future for addressing dual coinfections and combatting antimicrobial resistance. We eagerly anticipate commencing further studies and envisage publishing the next iteration of this research in the near future as we delve deeper into the implications and applications of our findings.

## Figures and Tables

**Table 1:** Specifics of the grid parameter data utilized.

AutoDock 4.2.6							
**Protein**	**Center Grid Box** **Dimensions (Å)**			**Spacing (Å)**	**Coordinates for the Center of the Grid Box**		
	*X*-axis	*Y*-axis	*Z*-axis	0.375	*X*-axis	*Y*-axis	*Z*-axis
MbtA	50	50	50		−2.439	19.515	32.895

**Table 2:** Comprehensive tabular overview of the top ten hits resulting from docking-based virtual screening of FDA library against MbtA receptor.

S. No.	Code	Structure	Compound Details	MOA and Therapeutic Application
1	a_617	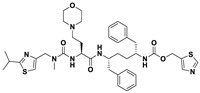	ZINC ID: ZINC000085537014 Cobicistat Formula: C_40_H_53_N_7_O_5_S_2_ Molar mass: 776.03 g·mol^−1^	Cobicistat (Tybost™) functions as a mechanism-based inhibitor of cytochrome P450 (CYP) 3A enzymes. It is approved in the EU as a pharmacokinetic enhancer, specifically a booster, of HIV-1 protease inhibitors (PIs) such as atazanavir and darunavir in adult patients [49]
2	a_391	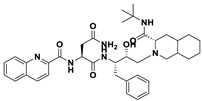	ZINC ID: ZINC000003914596 Saquinavir Formula: C_38_H_50_N_6_O_5_ Molar mass: 670.855 g·mol^−1^	Saquinavir was the first protease inhibitor developed for HIV therapy. It acts by cleaving viral polypeptide chains into functional proteins during the late stages of HIV replication [50]
3	a_821	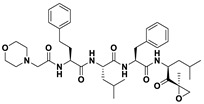	ZINC ID: ZINC000049841054 Carfilzomib Formula: C_38_H_50_N_6_O_5_ Molar mass: 670.855 g·mol^−1^	Carfilzomib (formerly PR-171) is a novel epoxyketone-based irreversible proteasome inhibitor. It specifically targets the chymotrypsin-like activity of the proteasome, preventing its normal function and causing a buildup of proteins within the cancer cells [51].
4	a_827	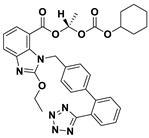	ZINC ID: ZINC000004074875 Candesartan Formula: C_24_H_20_N_6_O_3_ Molar mass: 440.463 g·mol^−1^	Candesartan blocks the vasoconstrictor and aldosterone-secreting effects of angiotensin II by selectively blocking the binding of angiotensin II to the AT1 receptor in many tissues, such as vascular smooth muscle and the adrenal gland [52].
5	a_85	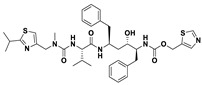	ZINC ID: ZINC000003944422 Ritonavir Formula: C_37_H_48_N_6_O_5_S_2_ Molar mass: 720.95 g·mol^−1^	Ritonavir is a protease inhibitor and is used as a booster with other protease inhibitors in the treatment of HIV infection. It prevents the proper maturation of the virus [53].
6	a_472	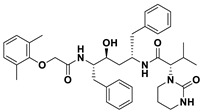	ZINC ID: ZINC000003951740 Lopinavir Formula: C_37_H_48_N_4_O_5_ Molar mass: 628.814 g·mol^−1^	Lopinavir is an antiretroviral medication primarily used in the treatment of HIV infection. It acts as a protease inhibitor in the final stages of the viral replication process thereby preventing the proper maturation of the virus, leading to immature and non-functional viral particles [53].
7	a_1276	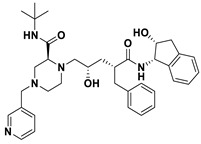	ZINC ID: ZINC000022448696 Indinavir Formula: C_36_H_47_N_5_O_4_ Molar mass: 613.803 g·mol^−1^	Indinavir is a protease inhibitor used as a component of highly active antiretroviral therapy to treat HIV/AIDS. It interferes with viral maturation, and contributes to the control of viral replication. It is also used as booster with other protease inhibitors [54].
8	a_1338	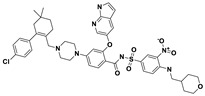	ZINC ID: ZINC000150338755 Venetoclax Formula: C_45_H_50_ClN_7_O_7_S Molar mass: 867.320 g·mol^−1^	Venetoclax is a B-cell lymphoma-2 (BCL-2) inhibitor. It binds to the BCL-2 protein, blocking its anti-apoptotic function
9	a_797	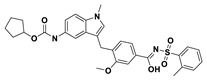	ZINC ID: ZINC000000896717 Accolate Formula: C_31_H_33_N_3_O_6_S Molar mass: 575.210 g·mol^−1^	Zafirlukast (Accolate) is a leukotriene receptor antagonist. It inhibits the activity of leukotrienes by binding to their receptors found on various cells in the airways, causing bronchodilation [55].
10	a_1388	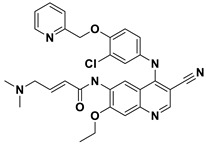	ZINC ID: ZINC000003916214 Neratinib Formula: C_30_H_29_ClN_6_O_3_ Molar mass: 556.200 g·mol^−1^	Neratinib is a tyrosine kinase inhibitor used in the treatment of certain types of breast cancers (targeted therapy). It works by irreversibly inhibiting multiple receptor tyrosine kinases, including HER2, epidermal growth factor receptor (EGFR), and HER4 [56].

**Table 3:** Detailed tabular representation of top 10 hits obtained through docking-based virtual screening from FDA library.

S. No	Ligand	Runs	Binding Energy (kcal/mol)	ESTAT	HB	VDW	DSOLV
1	a_617	200	−16.69	−0.9438	−2.5163	−23.9398	5.5597
2	a_391	200	−16.33	−0.6982	−2.18	−21.8025	4.9488
3	a_821	200	−16.08	−1.1546	−1.562	−23.9542	5.2432
4	a_827	200	−15.82	−0.8058	−1.9775	−21.7725	4.9785
5	a_85	200	−14.84	−0.3628	−2.1603	−20.6416	4.0052
6	a_472	200	−14.82	−0.8465	−2.6141	−20.8497	5.0782
7	a_1276	200	−14.80	−0.9278	−2.6608	−20.6792	5.1776
8	a_1338	200	−14.68	−1.3963	−1.6507	−27.4664	6.791
9	a_797	200	−14.50	−0.0758	−1.9036	−22.3757	4.7207
10	a_1388	200	−14.14	−0.0544	−1.7261	−21.6482	4.6123

**Table 4:** This table details hydrogen bonding and hydrophobic interactions of the top ten ligands in MbtA’s active site residues.

Sl. No.	Code	Docking Interactions with Active Site Amino Acid Residues	H-Bond Distance (Å)
1	a_617	H-bond-Asn258, Thr462, and Arg451 Hydrophobic-Val448, Asp436, Lys332, Phe353, Gly450, Gly330, Gly354, Leu360, Val352, Phe259, Cys263, Leu253, Ser213, Glu461, Ala356, Gly460, His257, Gly214, Met355, Thr216, Glu357, and Cys457	2.80, [3.06, 2.98], and [3.15, 2.48, 3.10]
2	a_391	H-bond-Asn258, Thr462, Arg451, Gly460, Ala356, and Phe259 Hydrophobic-His257, Leu253, Glu461, Val302, Leu360, Val352, Gly354, Gly329, Gln376, Ser331, Gly330, Phe353, Lys332, Val448, Met355, Thr216, Glu357, Val212, Gly214, and Ser213	2.87, 2.81, [3.23, 3.21], 3.05, 2.92, and [2.87, 3.07]
3	a_821	H-bond-Gly330, Thr462, and Arg451 Hydrophobic-Ala254, His257, Glu461, His523, Leu253, Leu126, His129, Gly214, Ser213, Val212, Met355, Ala356, Ser434, Glu357, Tyr432, Thr216, Val352, Lys332, Ser331, Phe353, Gly354, Gly460, and Ala459	2.95, 3.21, and 2.76
4	a_827	H-bond-Asn258, Arg451, Asp436, Gly354, and His257 Hydrophobic-Val212, Thr462, Glu461, Ala356, Val352, Gly330, Phe259, Leu360, Ser331, Phe353, Ser434, Tyr432, Met355, Tyr415, Gly214, Glu357, Thr216, and Ser213	[2.82, 2.96], [3.16, 3.06], 2.72, 3.01, and 2.87
5	a_85	H-bond-Ala356, Val212, Gly354, and His257 Hydrophobic-Gly214, Val352, Gly330, Met355, Thr462, Phe259, Asn258, Pro260, Leu253, Phe353, Val302, Ser331, Gly460, Glu357, Leu126, Glu461, Arg451, Ser213, Val448, Cys457, and Asp436	2.88, 2.80, 2.96, and 3.08
6	a_472	H-bond-Gly354, Gly460, Thr462, and Arg451 Hydrophobic-Asp436, Val448, Phe353, Met355, Lys332, Gly330, Ser331, Val352, Phe259, Leu360, Gly214, Val212, His129, Ser213, Leu126, Ala356, Glu461, Ala459, His257, and Leu253	2.75, 2.92, 3.16, and 2.86
7	a_1276	H-bond-Gly354, and Arg451 Hydrophobic-Asp436, Phe353, Leu360, Phe259, Gly329, Val352, Asn258, Glu461, Val302, Gly330, His257, Ala254, Thr462, Leu253, Gly460, Ala356, Glu357, Tyr432, Thr216, Gly214, and Ser434	2.66 and [2.63, 2.93]
8	a_1338	H-bond-Val352, Thr462, and Phe259 Hydrophobic-Gly460, Met355, Ser434, Tyr432, Arg451, Asp436, Cys457, Ile456, Gly450, Val455, Glu493, Glu334, Ser331, Val448, Lys332, Gly330, Phe353, Gly354, Gly329, Asn258, His257, Ala356, and Leu360	3.17, 2.83, and 3.15
9	a_797	H-bond-Asn258, His257, and Arg451 Hydrophobic-Glu461, Gly214, Gly460, Ser213, Thr462, Val212, Lys332, Asp436, Ser331, Cys457, Gly450, Val448, Phe353, Gly354, Val352, Gly330, Cys263, Leu360, Phe259, and Ala356	3.07, 3.04, and [2.87, 2.88]
10	a_1388	H-bond-His257, Phe259, and Thr462 Hydrophobic-Arg451, Val352, Phe353, Gly329, Ser331, Val448, Gln376, Lys332, Gly330, Asp436, Met355, Gly354, Ser213, Gly214, Glu357, Ala356, Gly256, Pro260, Gly460, Val212, Glu461, Val302, Leu253, and Asn258	2.88, 2.94, and 2.94

## Data Availability

Data are contained within the article and supplementary materials. Also, the datasets generated during and/or analyzed during the current study are available from the corresponding author upon reasonable request.

## Supplementary Materials

The following supporting information can be downloaded at: https://www.mdpi.com/article/10.3390/pathogens12121433/s1, Figure S1:The docked confirmation of (a) a_617, (b) a_391, (c) a_821, (d) a_827, (e) a_85, (f) a_472, (g) a_1276, (h) a_1338, (i) a_797, and (j) a_1388 in the active site of MbtA highlighting various interactions. In the plot, hydrophobic interactions are depicted as maroon spiked arcs, while hydrogen bonding interactions are shown as dashed green lines, with their lengths indicated in Å. The color scheme distinguishes various atoms and bonds: carbon atoms are black, oxygen atoms are red, sulphur as yellow, and nitrogen atoms are blue. Amino acid residue bonds are represented in brown, and ligands are colored violet.; Figure S2: Interactions made by the ligands (a) a_617: Cobicistat, (b) a_391: Saquinavir, (c) a_821: Carfilzomib, (d) a_827: Candesartan, (e) a_85: Ritonavir, (f) a_472: Lopinavir, and (g) a_1276: Indinavir in complex with MbtA for the most stable frame during MD simulation as evidenced from RMSD graph. In the plot, hydrophobic interactions are depicted as maroon spiked arcs, while hydrogen bonding interactions are shown as dashed green lines, with their lengths indicated in Å. The color scheme distinguishes various atoms and bonds: carbon atoms are black, oxygen atoms are red, sulfur as yellow, and nitrogen atoms are blue. Amino acid residue bonds are represented in brown, and ligands are colored violet; Figure S3: Graphical representation of the comparison between the conformations of the apo-protein and the protein-ligand complexes: (a) a_617: Cobicistat-MbtA, (b) a_391: Saquinavir-MbtA, (c) a_821: Carfilzomib-MbtA, (d) a_827: Candesartan-MbtA, (e) a_85: Ritonavir-MbtA, (f) a_472: Lopinavir-MbtA, and (g) a_1276: Indinavir-MbtA. For a particular image: (i) left: superimposed stable confirmations of apo-protein and PLC at a particular stable time frame [golden: apo-protein and cyan: protein-ligand complex], (ii) middle: 2D maps of residue-residue distances and standard deviation, and (iii) right: 2D maps of residue-residue differences; Figure S4: Principal component analysis of individual protein–ligand complexes: the collective motion of (a) a_617: Cobicistat, (b) a_391: Saquinavir, (c) a_821: Carfilzomib, (d) a_827: Candesartan, (e) a_85: Ritonavir, (f) a_472: Lopinavir, and (g) a_1276: Indinavir with MbtA using projections of MD trajectories on five various eigenvectors (1_2dproj: black, 2_2dproj: red, 3_2dproj: red, 4_2dproj: blue); Figure S5: Root mean square fluctuation (RMSF) nm of eigenvectors-backbone highlighting the stability of the protein in the presence of ligand (a) a_617: Cobicistat (blue), (b) a_391: Saquinavir (red), (c) a_821: Carfilzomib (yellow), (d) a_827: Candesartan (brown), (e) a_85: Ritonavir (black), (f) a_472: Lopinavir (green), and (g) a_1276: Indinavir (turquoise); Figure S6: Principal component analysis of protein-ligand complexes: the collective motion along with the Root mean square fluctuation (RMSF) nm of first two eigenvectors fluctuations (vector 1 and vector 2), eigenvector-backbone highlighting the stability of the protein in the presence of ligand (a) a_617: Cobicistat (yellow), (b) a_391: Saquinavir (green), (c) a_821: Carfilzomib (brown), (d) a_827: Candesartan (magenta), (e) a_85: Ritonavir (red), (f) a_472: Lopinavir (blue), and (g) a_1276: Indinavir (black) with MbtA using projections of MD trajectories on two eigenvectors corresponding to the first two principal components.
