# Supplementary material for: In Silico Drug Repurposing Studies for the Discovery of Novel Salicyl-AMP Ligase (MbtA)Inhibitors
Source: Pathogens. 2023 Dec 9;12(12):1433. doi: 10.3390/pathogens12121433 (PMC10745912; doi:10.3390/pathogens12121433)
Supplement: Supplementary file 1 [file pathogens-12-01433-s001.zip › pathogens-2658916-supplementary.pdf]

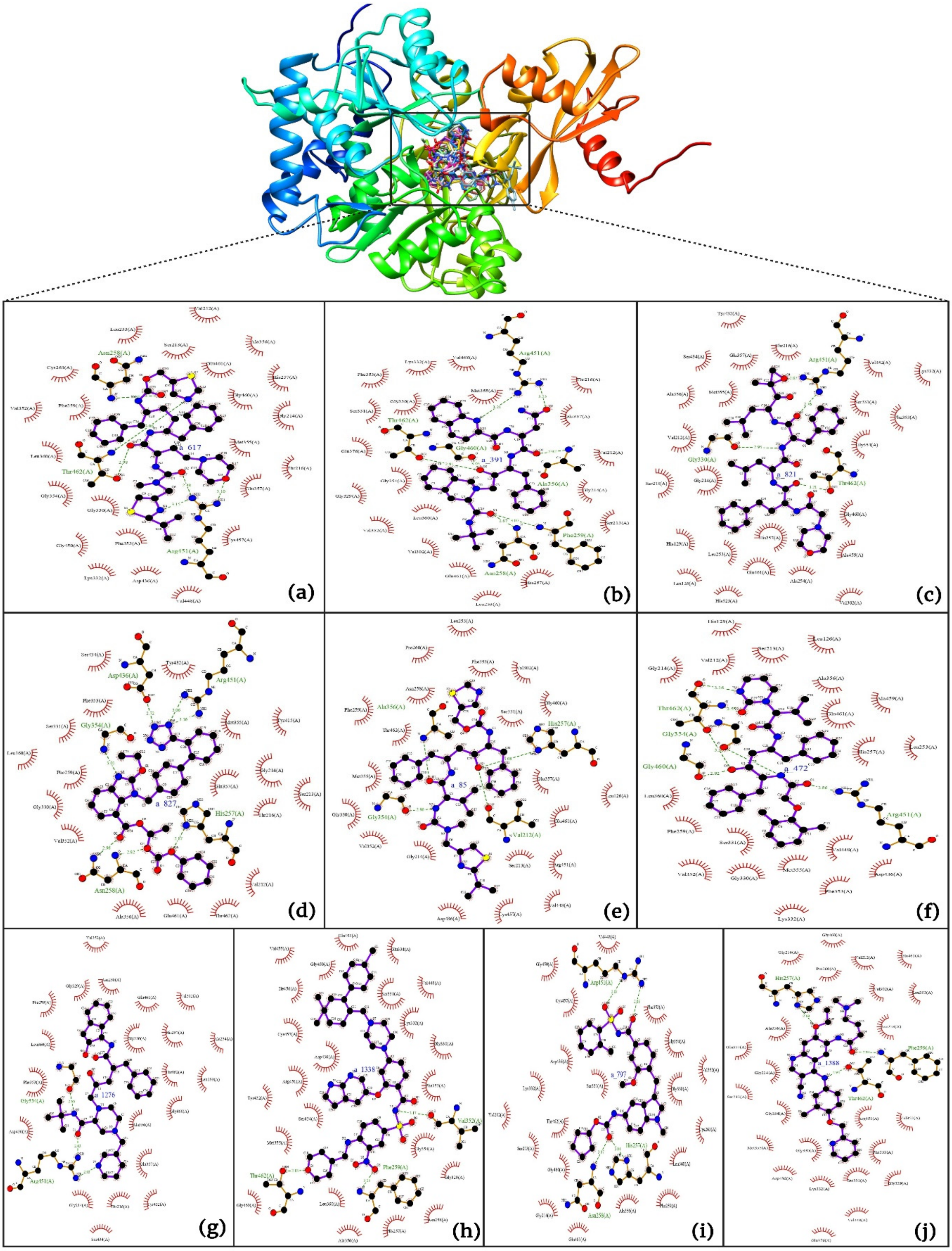


**Figure S1.** The docked confirmation of (a) a_617, (b) a_391, (c) a_821, (d) a_827, (e) a_85, (f) a_472, (g) a_1276, (h) a_1338, (i) a_797, and (j) a_1388 in the active site of MbtA highlighting various interactions. In the plot, hydrophobic interactions are depicted as maroon spiked arcs, while hydrogen bonding interactions are shown as dashed green lines, with their lengths indicated in Å. The color scheme distinguishes various atoms and bonds: carbon atoms are black, oxygen atoms are red, sulphur as yellow, and nitrogen atoms are blue. Amino acid residue bonds are represented in brown, and ligands are colored violet.

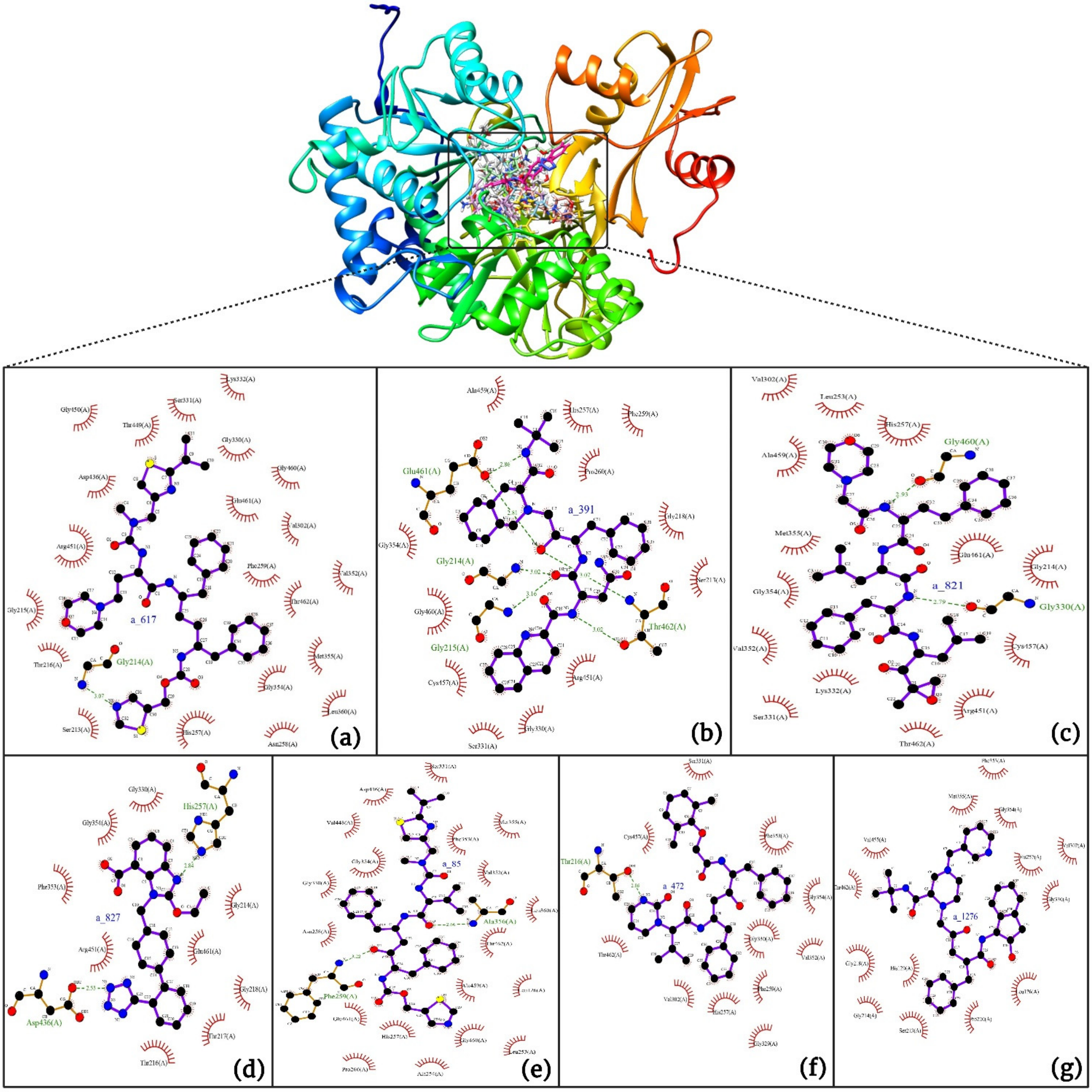


**Figure S2.** Interactions made by the ligands (a) a_617: Cobicistat, (b) a_391: Saquinavir, (c) a_821: Carfilzomib, (d) a_827: Candesartan, (e) a_85: Ritonavir, (f) a_472: Lopinavir, and (g) a_1276: Indinavir in complex with MbtA for the most stable frame during MD simulation as evidenced from RMSD graph. In the plot, hydrophobic interactions are depicted as maroon spiked arcs, while hydrogen bonding interactions are shown as dashed green lines, with their lengths indicated in Å. The color scheme distinguishes various atoms and bonds: carbon atoms are black, oxygen atoms are red, sulfur as yellow, and nitrogen atoms are blue. Amino acid residue bonds are represented in brown, and ligands are colored violet

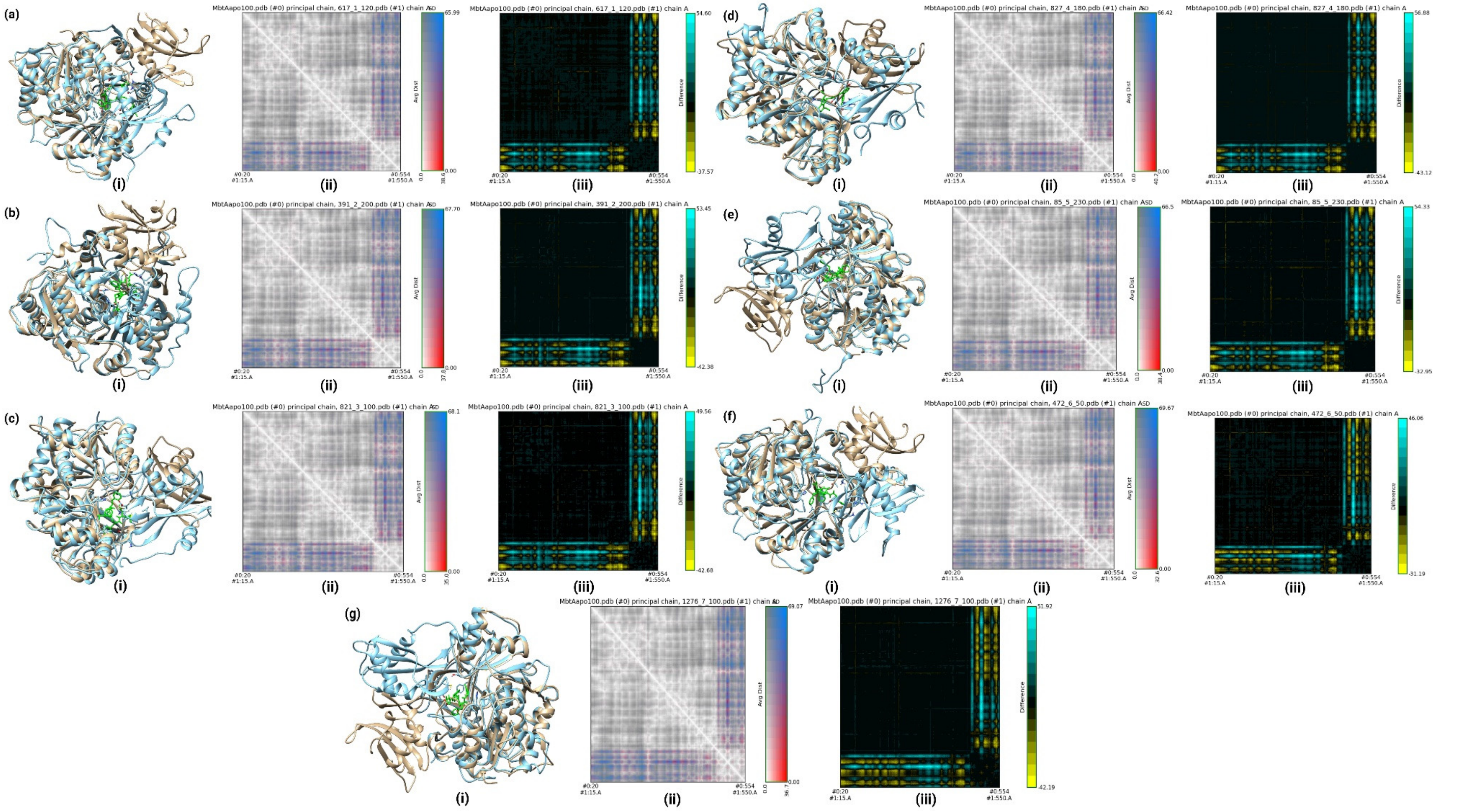


**Figure S3.** Graphical representation of the comparison between the conformations of the apo-protein and the protein-ligand complexes: (a) a_617: Cobicistat-MbtA, (b) a_391: Saquinavir-MbtA, (c) a_821: Carfilzomib-MbtA, (d) a_827: Candesartan-MbtA, (e) a_85: Ritonavir-MbtA, (f) a_472: Lopinavir-MbtA, and (g) a_1276: Indinavir-MbtA. For a particular image: (i) left: superimposed stable confirmations of apo-protein and PLC at a particular stable time frame [golden: apo-protein and cyan: protein-ligand complex], (ii) middle: 2D maps of residue-residue distances and standard deviation, and (iii) right: 2D maps of residue-residue differences.

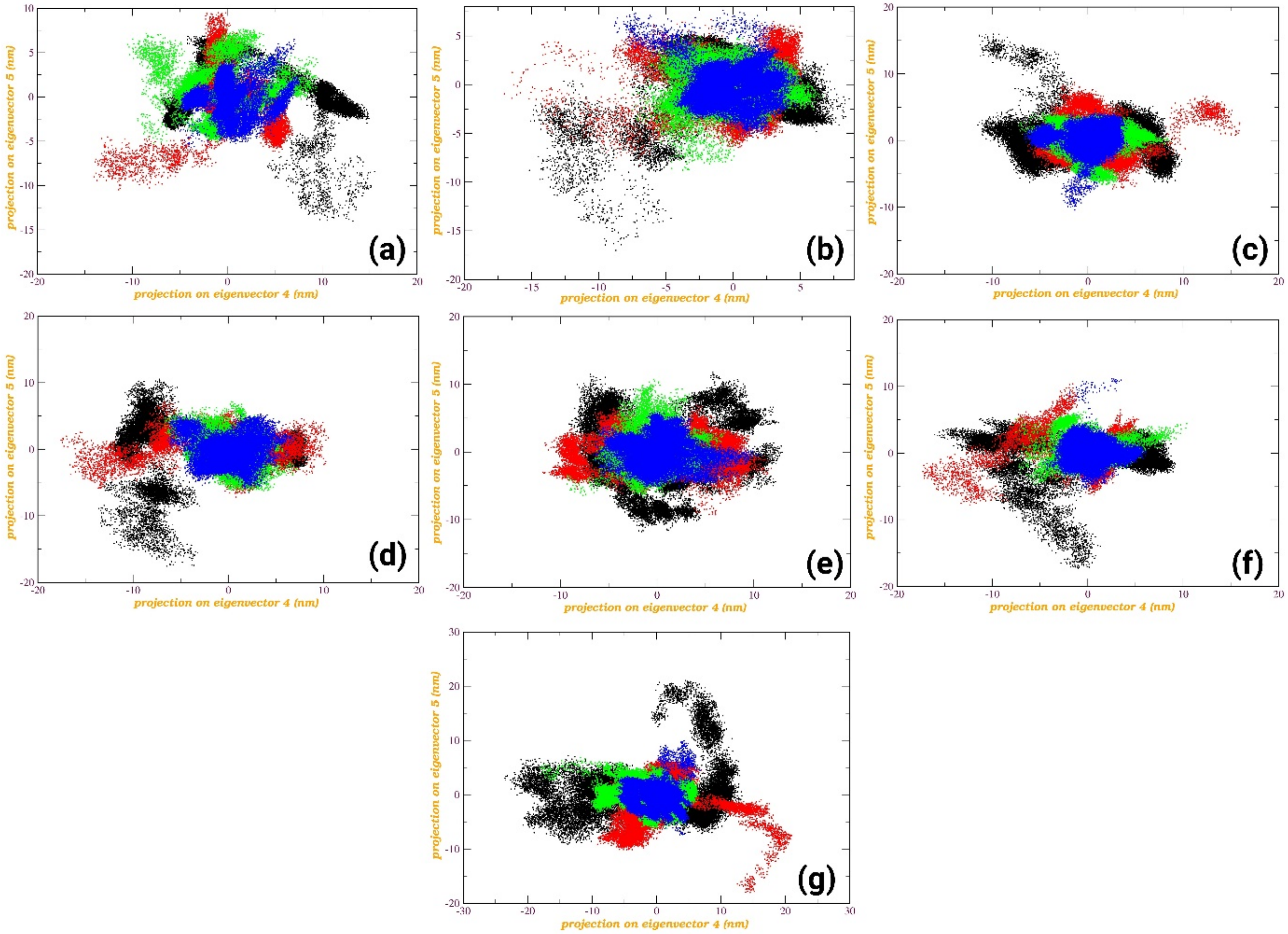


**Figure S4.** Principal component analysis of individual protein–ligand complexes: the collective motion of (a) a_617: Cobicistat, (b) a_391: Saquinavir, (c) a_821: Carfilzomib, (d) a_827: Candesartan, (e) a_85: Ritonavir, (f) a_472: Lopinavir, and (g) a_1276: Indinavir with MbtA using projections of MD trajectories on five various eigenvectors (1_2dproj: black, 2_2dproj: red, 3_2dproj: red, 4_2dproj: blue).

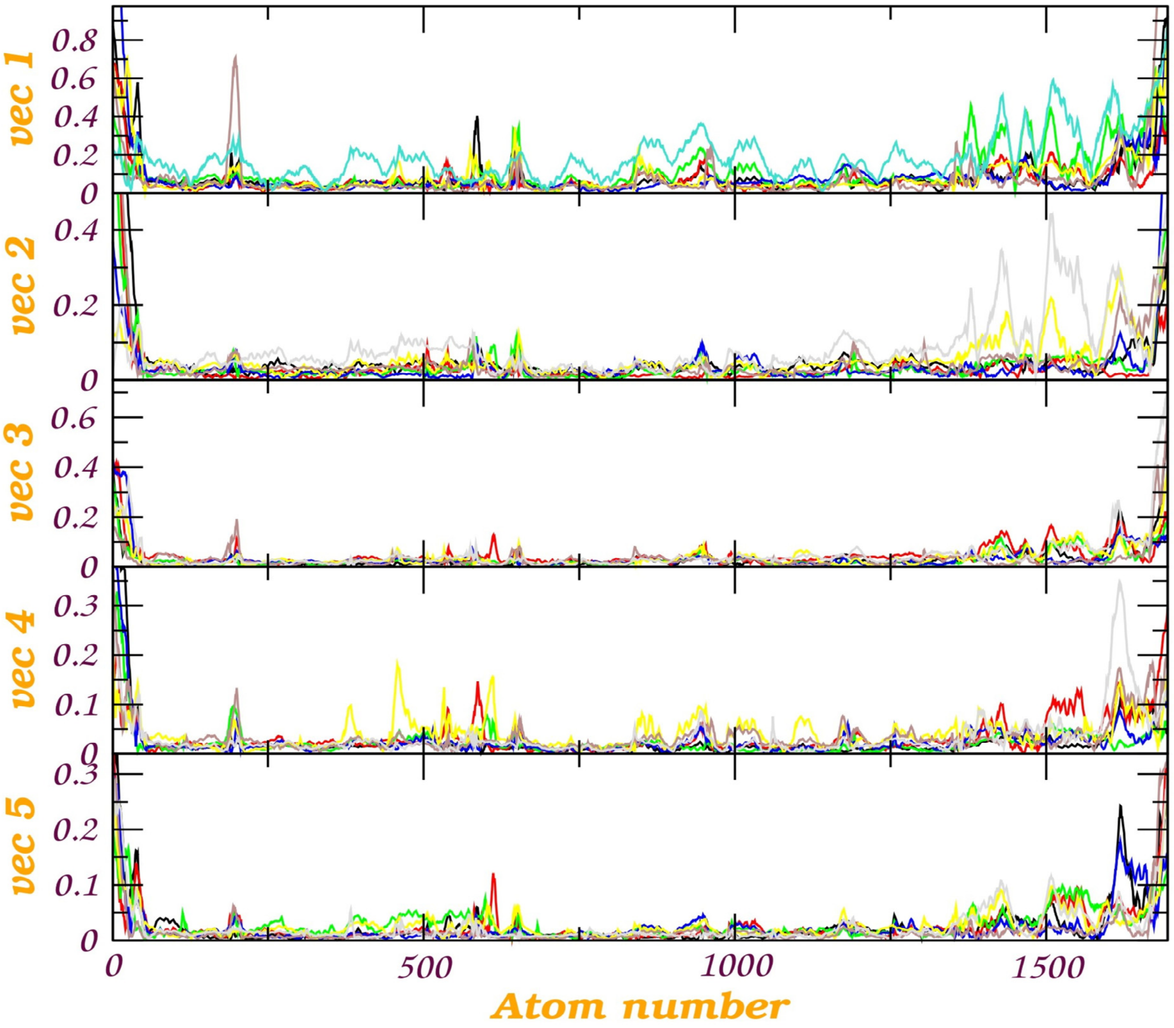


**Figure S5.** Root mean square fluctuation (RMSF) nm of eigenvectors-backbone highlighting the stability of the protein in the presence of ligand (a) a_617: Cobicistat (blue), (b) a_391: Saquinavir (red), (c) a_821: Carfilzomib (yellow), (d) a_827: Candesartan (brown), (e) a_85: Ritonavir (black), (f) a_472: Lopinavir (green), and (g) a_1276: Indinavir (turquoise).

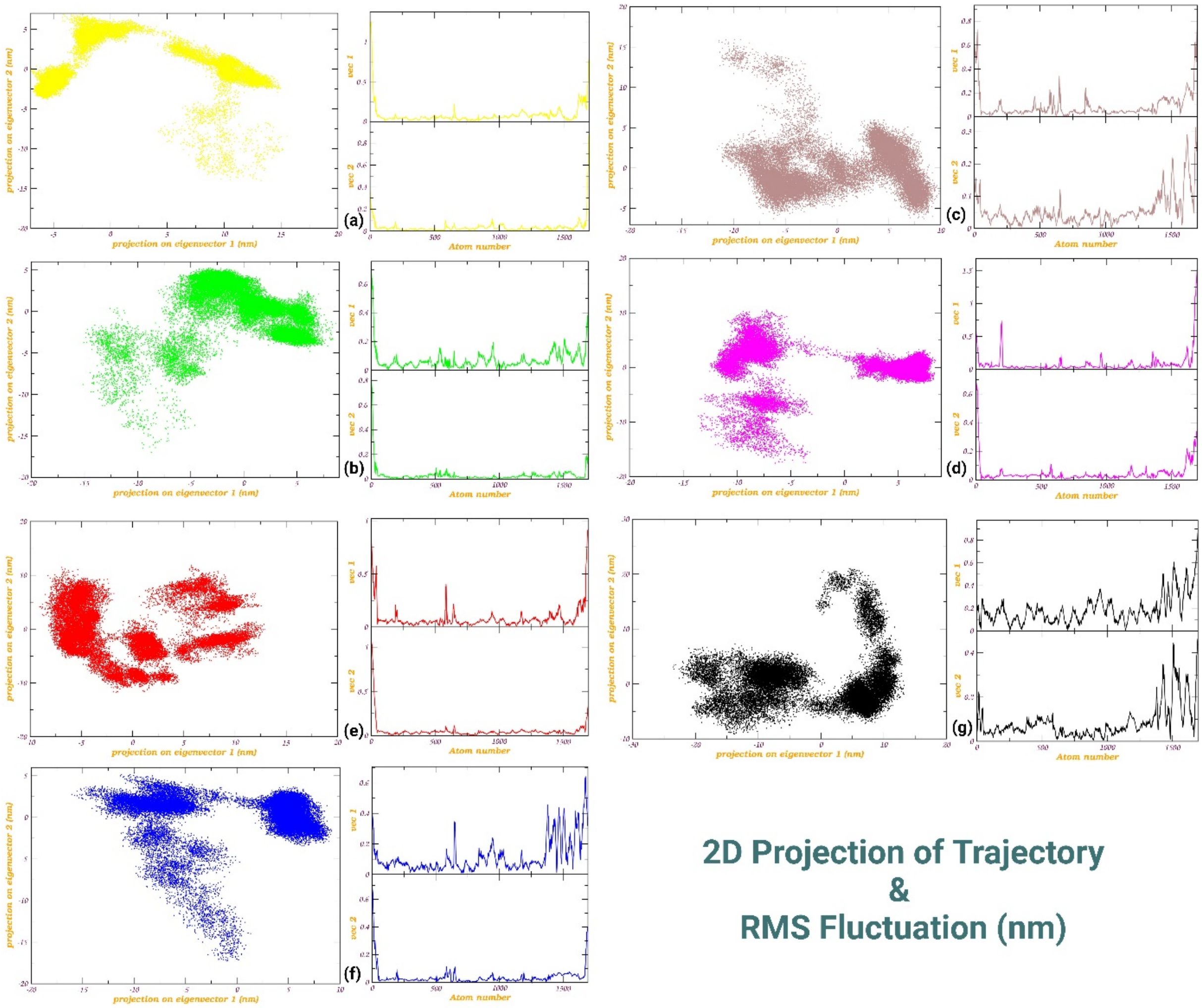


**Figure S6.** Principal component analysis of protein-ligand complexes: the collective motion along with the Root mean square fluctuation (RMSF) nm of first two eigenvectors fluctuations (vector 1 and vector 2), eigenvector-backbone highlighting the stability of the protein in the presence of ligand (a) a_617: Cobicistat (yellow), (b) a_391: Saquinavir (green), (c) a_821: Carfilzomib (brown), (d) a_827: Candesartan (magenta), (e) a_85: Ritonavir (red), (f) a_472: Lopinavir (blue), and (g) a_1276: Indinavir (black) with MbtA using projections of MD trajectories on two eigenvectors corresponding to the first two principal components.
